# Respiratory *Bordetella bronchiseptica* Carriage is Associated with Broad Phenotypic Alterations of Peripheral CD4^+^CD25^+^ T Cells and Differentially Affects Immune Responses to Secondary Non-Infectious and Infectious Stimuli in Mice

**DOI:** 10.3390/ijms19092602

**Published:** 2018-09-01

**Authors:** Andreas Jeron, Julia D. Boehme, Julia Volckmar, Marcus Gereke, Tetyana Yevsa, Robert Geffers, Carlos A. Guzmán, Jens Schreiber, Sabine Stegemann-Koniszewski, Dunja Bruder

**Affiliations:** 1 Infection Immunology Group, Institute of Medical Microbiology, Infection Control and Prevention, Health Campus Immunology, Infectiology and Inflammation, Otto-von-Guericke University Magdeburg, 39120 Magdeburg, Germany; Andreas.Jeron@med.ovgu.de (A.J.); Julia.Boehme@helmholtz-hzi.de (J.D.B.); Julia.Volckmar@helmholtz-hzi.de (J.V.); Marcus.Gereke@helmholtz-hzi.de (M.G.); Sabine.Stegemann-Koniszewski@med.ovgu.de (S.S.-K.); 2 Immune Regulation Group, Helmholtz Centre for Infection Research, 38124 Braunschweig, Germany; 3 Department of Vaccinology and Applied Microbiology, Helmholtz Centre for Infection Research, 38124 Braunschweig, Germany; Tetyana.Yevsa@helmholtz-hzi.de (T.Y.); CarlosAlberto.Guzman@helmholtz-hzi.de (C.A.G.); 4 Genome Analytics Group, Helmholtz Centre for Infection Research, 38124 Braunschweig, Germany; Robert.Geffers@helmholtz-hzi.de; 5 Experimental Pneumology, University Hospital for Pneumology, Health Campus Immunology, Infectiology and Inflammation, Otto-von-Guericke University Magdeburg, 39120 Magdeburg, Germany; Jens.Schreiber@med.ovgu.de

**Keywords:** respiratory microbial carriage, polymicrobial interactions, respiratory immune regulation, secondary infections, vaccination, regulatory T cells, influenza A virus, *Bordetella bronchiseptica*, *Listeria monocytogenes*

## Abstract

The respiratory tract is constantly exposed to the environment and displays a favorable niche for colonizing microorganisms. However, the effects of respiratory bacterial carriage on the immune system and its implications for secondary responses remain largely unclear. We have employed respiratory carriage with *Bordetella bronchiseptica* as the underlying model to comprehensively address effects on subsequent immune responses. Carriage was associated with the stimulation of *Bordetella*-specific CD4^+^, CD8^+^, and CD4^+^CD25^+^Foxp3^+^ T cell responses, and broad transcriptional activation was observed in CD4^+^CD25^+^ T cells. Importantly, transfer of leukocytes from carriers to acutely *B. bronchiseptica* infected mice, resulted in a significantly increased bacterial burden in the recipient’s upper respiratory tract. In contrast, we found that respiratory *B. bronchiseptica* carriage resulted in a significant benefit for the host in systemic infection with *Listeria monocytogenes.* Adaptive responses to vaccination and influenza A virus infection, were unaffected by *B. bronchiseptica* carriage. These data showed that there were significant immune modulatory processes triggered by *B. bronchiseptica* carriage, that differentially affect subsequent immune responses. Therefore, our results demonstrated the complexity of immune regulation induced by respiratory bacterial carriage, which can be beneficial or detrimental to the host, depending on the pathogen and the considered compartment.

## 1. Introduction

The lung as the central site of gas exchange is, together with the conducting airways, constantly exposed to all kinds of environmental factors and thereby confronted with potential hazards, such as pollutants and pathogenic microorganisms. While it was long believed to represent a sterile compartment, research of the last years has delivered evidence that the lower respiratory tract displays a favorable niche for commensal microorganisms [1,2]. The lungs central role in oxygen supply, its delicate structure, its constant exposure to harmful as well as harmless microorganisms, and its potential to serve as an entry site for invading pathogens, altogether demand for an effective and at the same time tightly regulated immune system.

Immunity in the lung is conferred through the combined action of different types of sentinel cells, such as airway and alveolar epithelial cells, alveolar macrophages (AM), and dendritic cells (DC), which surveil the respiratory tract and in the case of pathogen encounter induce well-orchestrated innate and adaptive responses, involving multiple cell-types and mediators [3,4,5,6]. To protect from overshooting and harmful responses, sophisticated regulatory mechanisms are in place. These can create an immune regulatory environment and can suppress innate and adaptive immune cell function through several mechanisms, such as the production of anti-inflammatory mediators or direct cell-to-cell interactions [3,4,7,8]. However, next to protecting the lung from the detrimental effects of host immunity, these regulatory mechanisms can in turn favor the persistence of colonizing microorganisms and frequently display targets of microbial evasion strategies [9,10,11].

Given its function, as well as its anatomic exposition and resulting immunological features, the respiratory tract harbors an exceptional challenge regarding the regulation of immune responses towards multiple antigens simultaneously present. These can be polymicrobial interactions, such as co-infections with different respiratory pathogens, the co-occurrence of commensal and pathogenic bacteria, or interactions between respiratory infections and non-infectious stimuli, such as inhaled allergens [12,13,14]. At the same time, there is an increasing awareness of immune regulatory effects across compartments to remote sites, which to date are most appreciated for the gut microbiome [13,15,16]. Nevertheless, there is also evidence for inflammatory processes in the respiratory tract to influence systemic immunity [13,17,18,19]. While we are only beginning to understand these multi-faceted interactions, the underlying mechanisms to date remain mostly elusive.

*Bordetella bronchiseptica* is a gram-negative respiratory pathogen of several mammalian species, which is causing a number of veterinary respiratory syndromes in domestic as well as agriculturally important and food-producing animals, whilst humans are only rarely infected [20,21]. *B. bronchiseptica* is genetically closely related to the major human pathogen *Bordetella pertussis*, the cause of whooping cough. Both *B. bronchiseptica* and *B. pertussis* regulate the expression of several virulence factors, including toxins and a type III secretion system (T3SS), by a two-component system that controls phenotypic modulation [21,22]. Due to the lack of *B. pertussis* models in commonly used laboratory animal species and its close relatedness to *B. bronchiseptica*, the latter is frequently utilized as an animal model to explore mechanisms of *B. pertussis* pathogenesis (reviewed in [21]). Nevertheless, there are fundamental differences namely that *B. bronchiseptica* can persist in the respiratory tract of its hosts and is able to cause chronic infections [20,23], whereas in humans there is no evidence of prolonged *B. pertussis* carriage [24]. *B. bronchiseptica* interacts with the host immune system in multiple ways, affecting a variety of immune cells. Both intracellular survival [25,26], as well as clear cytotoxic effects on macrophages, have been described for *B. bronchiseptica* [27,28,29]. Importantly, *B. bronchiseptica* is able to modulate cytokine production by macrophages and DC through its T3SS, with clear consequences for subsequent T cell responses [30,31]. Furthermore, it has been described that *B. bronchiseptica* invades and survives in mouse DC in vitro [32], and that nasal persistence affects DC dynamics in the upper and lower respiratory tract [33]. In vivo, *B. bronchiseptica* induces a strong influx of neutrophils, monocytes/macrophages, and lymphocytes into the lungs [20]. Furthermore, the T3SS of *B. bronchiseptica* was shown to drive DC migration to the secondary lymphoid tissue, which was observed together with a strong induction of anti-inflammatory interleukin (IL) −10 and a down-regulation of the interferon (IFN)-γ response [34,35,36]. Based on these findings, the induction of an immune suppressive T_reg_ response during *B. bronchiseptica* carriage, as a mechanism of immune evasion has been suggested, but to our knowledge this was not addressed in detail [35,36].

In our study, we aimed at assessing the regulatory potential of respiratory microbial carriage on secondary immune responses, both in the lung and the periphery. We took advantage of *B. bronchiseptica’s* natural potential to colonize the host [21] and chose it as a model for respiratory carriage. In this model, we have comprehensively elucidated its effects on local and peripheral immune responses to different secondary stimuli. For these secondary immunological challenges, we employed models of both respiratory and systemic infection with viral and bacterial pathogens, as well as vaccination with a model antigen. Furthermore, our study included the analysis of the peripheral CD4^+^ T cell pool on the functional and gene transcriptional level during acute *B. bronchiseptica* infection, as well as during subsequent carriage. We show for the first time that clear and broad changes in the peripheral T cell pool, including the induction of pathogen-specific T_reg_, are mediated by *B. bronchiseptica* carriage. Furthermore, our study reveals significant immune regulatory processes triggered by *B. bronchiseptica* carriage, which differentially affect secondary immune responses.

## 2. Results

### 2.1. B. bronchiseptica Establishes Respiratory Tract Carriage Despite the Presence of Pathogen-Specific T Cells in Mice

**Figure 1:**
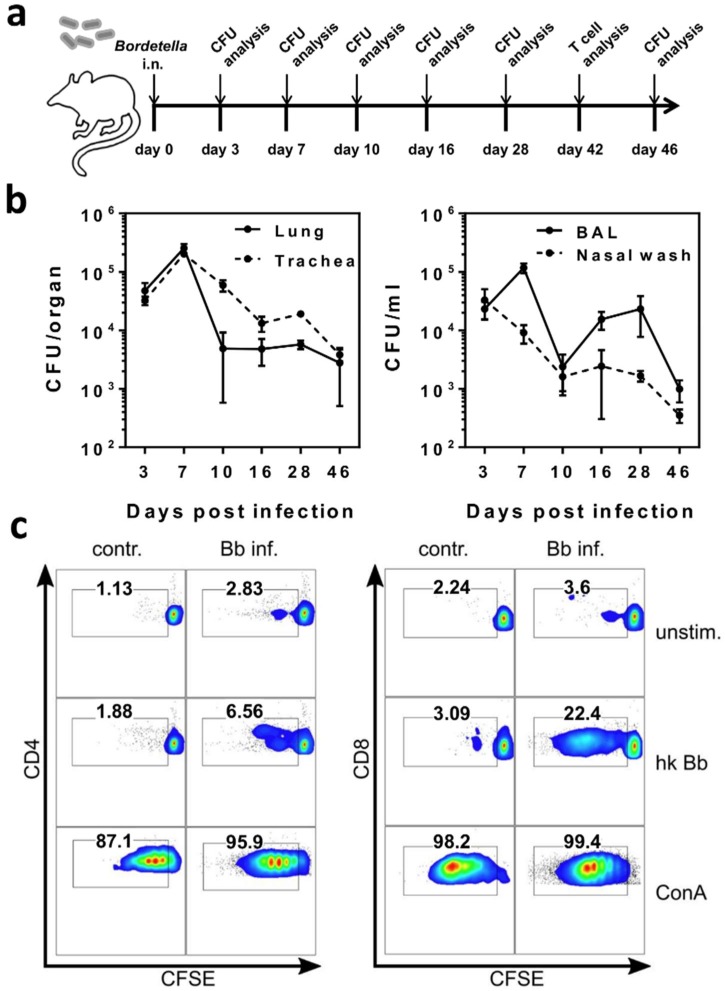
*B. bronchiseptica* establishes respiratory tract carriage, despite the presence of pathogen-specific T cells in mice. Wild type BALB/c mice were intranasally infected with 5 × 10^5^ CFU *B. bronchiseptica* (Bb). (**a**) Experimental setup. (**b**) Mice were sacrificed at the indicated time points, and the CFU load was determined in homogenized lung and tracheal tissue, as well as in bronchoalveolar lavage (BAL) and nasal wash. Data represent the mean ± SEM of three mice per time point. (**c**) On day 42 post infection, splenocytes were isolated from three *B. bronchiseptica* (Bb) infected mice per group, pooled, carboxyfluorescein diacetate succinimidyl ester (CFSE) labelled, and re-stimulated in vitro, with or without heat-killed bacteria at a Bb-to-cell ratio of 10:1 for 4 days (2 × 10^6^ splenocytes/well). Concanavalin A was added as a positive control. Cells were analyzed by flow cytometry, for CFSE loss in the CD4^+^ (**left**) and CD8^+^ (**right**) T cell compartment. Data are representative for one out of two independent experiments.

**Figure 2:**
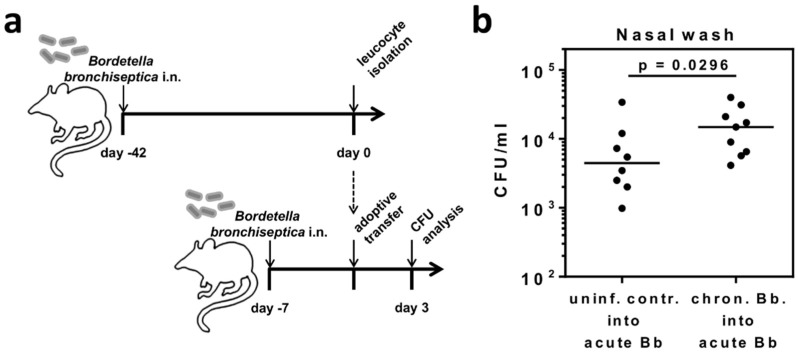
Transfer of leukocytes isolated from *B. bronchiseptica* carriers interferes with anti-bacterial control in acutely infected recipients. Donor BALB/c mice were infected with *B. bronchiseptica* and six weeks later leukocytes were isolated from spleen, bronchial, and cervical lymph nodes, followed by B220-marker-depletion. Leukocytes were injected intravenously into recipients likewise infected with *B. bronchiseptica* seven days before transfer. (**a**) Experimental setup. (**b**) On day 10 post infection (day 3 post transfer) recipient mice were sacrificed and the CFU burden in the nasal wash was determined. Data and the median/group are shown for individual mice and were compiled from two independent experiments. Groups were compared by unpaired, one-sided Mann-Whitney test.

We chose *B. bronchiseptica* as an experimental infection model to study the effects of chronic microbial carriage on immune regulatory mechanisms, and on subsequent immunological challenges in the lung and in the periphery. In our mouse model, *B. bronchiseptica* was still detectable in the lung, trachea, bronchoalveolar space, and nasal cavity, seven weeks following intranasal infection with 5 × 10^5^ CFU. In all compartments, peak bacterial loads were detected 7 days post infection, followed by respiratory carriage of *B. bronchiseptica* with reduced bacterial numbers from day 10 onwards (Figure 1a,b). *B. bronchiseptica*-specific lymphocyte proliferation was detectable in cells isolated from the cervical lymph nodes from day 16 post infection, and in splenocytes by day 46 post infection (data not shown). As our aim was to address the regulatory potential of respiratory microbial carriage, after acute infection and independent of the primary adaptive immune response, we chose late time points of 42 days or longer—post infection for our analyses. Importantly, due to the presence of Bordetella-specific CD4^+^, as well as CD8^+^ T cells in the periphery, a completed specific adaptive T cell response was confirmed during bacterial carriage (Figure 1c). Immune regulatory effects of *B. bronchiseptica* involving multiple cell types of the immune system have been described in References [25,26,27,28,29,30,31]. Therefore, we hypothesized that inefficient bacterial clearance from the respiratory tract was the potential consequence of Bordetella-induced alterations of host immunity, possibly associated with the induction of immunosuppressive cells. To experimentally address this hypothesis, we adoptively transferred B-cell depleted leukocytes from 6-week *B. bronchiseptica* infected mice into recipient mice, which had been infected with *B. bronchiseptica* one week before (Figure 2a). To assess potentially suppressive effects of the transferred cells on the clearance of *B. bronchiseptica*, we determined the bacterial load in the upper respiratory tract of the recipient mice three days after transfer. Control of *B. bronchiseptica* growth was significantly less efficient in the nasal cavity of mice that received leukocytes from carriers, as compared to those that received cells from uninfected donors (Figure 2b). In conclusion, these data showed that *B. bronchiseptica* was able to cause respiratory carriage, despite the presence of pathogen-specific T cells and that leukocytes transferred from *B. bronchiseptica* carriers interfered with pathogen clearance in acute infection.

### 2.2. Respiratory B. bronchiseptica Carriage Is Associated with Broad Phenotypic Alterations of Peripheral CD4^+^CD25^+^ T Cells and the Induction of Pathogen-Specific Regulatory T Cells

**Figure 3:**
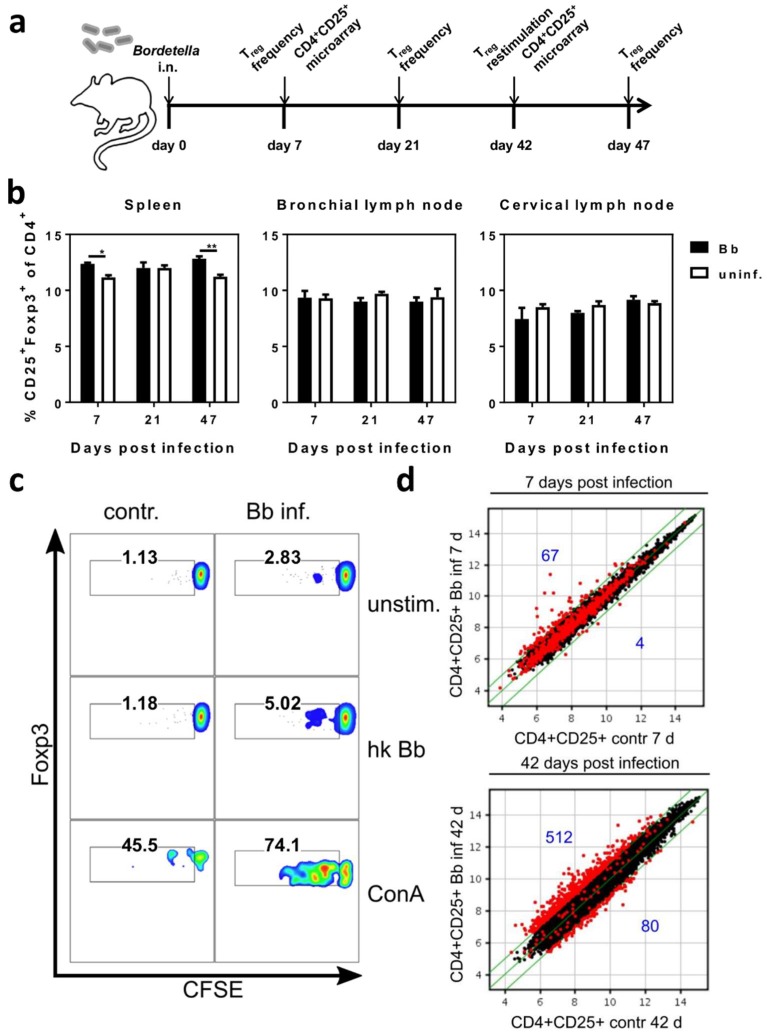
Respiratory *B. bronchiseptica* carriage is associated with broad phenotypic alterations of peripheral CD4^+^CD25^+^ T cells and induction of specific regulatory T cells. BALB/c mice were intranasally infected with 5 × 10^5^ CFU *B. bronchiseptica* (Bb) or treated with PBS. (**a**) Experimental setup. (**b**) Mice were sacrificed at the indicated time points and lymphocytes were isolated from the spleen, bronchial, and cervical lymph nodes, and analyzed for the frequency of CD25^+^Foxp3^+^ T_reg_ within the CD4^+^ T cell population by flow cytometry. Data represent the mean ± SEM of *n =* 4–5 mice per group. Groups were compared by two-way ANOVA with multiple comparisons, followed by Bonferroni’s post-test. * *p* = 0.012; ** *p* = 0.0017. (**c**) On day 42 post infection, splenocytes were isolated from groups of three mice per group, were pooled, carboxyfluorescein diacetate succinimidyl ester (CFSE) labelled, and re-stimulated in vitro, with or without heat-killed bacteria in a Bb-to-cell ratio of 10:1 for 4 days (2 × 10^6^ cells/well). Concanavalin A was added as a positive control. Cells were analyzed by flow cytometry, for CFSE loss of the CD4^+^CD25^+^Foxp3^+^ T_reg_ population. Data are representative for one out of two independent experiments with similar results. (**d**) Splenocytes from both infected and control mice were isolated and pooled (*n =* 6 per group) on day 7 or 42 post infection. CD4^+^CD25^+^ cells were flow cytometrically sorted for RNA preparation. Samples were analyzed on whole transcriptome microarrays. Data represent scatter plots of normalized log2 transformed signal intensities. For both the time points day 7 and day 42 post infection, the fold changes of transcripts were calculated for CD4^+^CD25^+^ T cells from infected mice vs. CD4^+^CD25^+^ T cells from uninfected controls. Red dots represent regulated genes with a fold change greater than ±2 on either of the analyzed time points. The numbers of up- and down-regulated genes are indicated in blue. Green lines indicate the fold change threshold.

**Figure 4:**
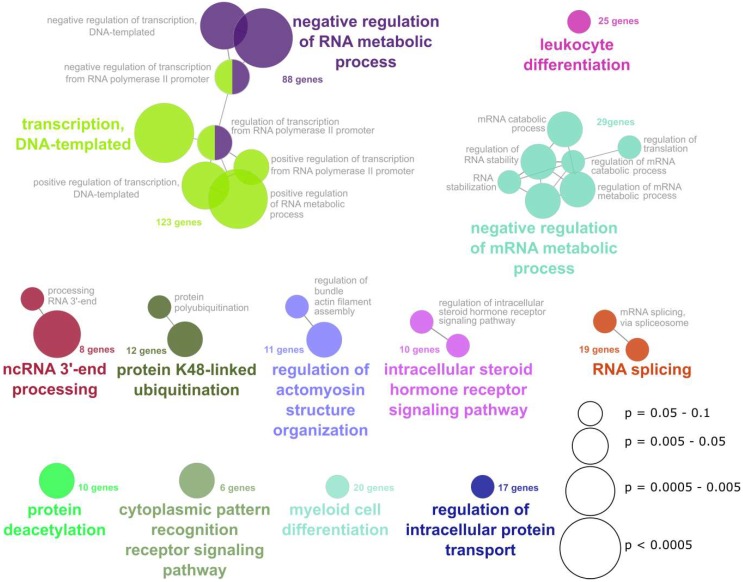
Gene ontology enrichment analysis of the genes regulated in CD4^+^CD25^+^ T cells isolated from *B. bronchiseptica* carriers. Transcripts that were regulated more than two-fold in CD4^+^CD25^+^ T cells on day 42 post-infection as compared to uninfected controls were analyzed for significant gene ontology (GO)-term enrichment (GO: biological process), with a right-sided hypergeometric test and Bonferroni step-down p-value correction (*p* < 0.1). GO-term levels were restricted from level 8 to 16 and at least five genes per GO-term were required. The analysis resulted in 13 significantly enriched GO-term groups (color coded). Enriched GO-terms were grouped and connected if they were >50% similar regarding their gene symbol content. The length of the displayed connecting edges and the resulting node distances between individual GO-terms (individual colored circles) within the 13 GO-term groups represent the gene content similarities (kappa-score) of the GO-terms shown. The circle size represents the enrichment *p*-value (see *p*-value legend). Each GO-term group is named according to the GO-term in the group that yielded the lowest enrichment *p*-value of all group members. The numbers of experimentally (microarray)-defined genes in the respective GO-term group is stated. Supplementary table S1 lists the displayed GO-term groups together with the fold-change regulation of the genes included in the respective group (top 10 up-and down-regulated).

The effect of the adoptive transfer of leukocytes from 6-week *B. bronchiseptica* infected mice to acutely infected mice, pointed towards the presence of immunosuppressive cells that impede the elimination of *B. bronchiseptica* from the respiratory tract. Furthermore, the induction of a T_reg_ response by *B. bronchiseptica* has been implicated in past studies [35,36]. Therefore, we characterized the peripheral CD4^+^ T cell pool at different time points following *B. bronchiseptica* infection (Figure 3a). First, we investigated the frequency of CD4^+^CD25^+^Foxp3^+^ T_reg_ in *B. bronchiseptica* infected mice, in comparison to control mice on days 7, 21, and 47, post infection (Figure 3b). Unexpectedly, T_reg_ frequencies were not changed over the course of infection. Of note, this was consistently the case in the draining (bronchial and cervical) lymph nodes, as well as in the periphery (spleen). At the same time, comparing T_reg_ frequencies between infected and control mice revealed a slight though significant reduction in the spleens, but not lymph nodes, of infected mice on day 7 and day 47. We furthermore analyzed the presence of *B. bronchiseptica*-specific CD4^+^CD25^+^Foxp3^+^ T_reg_ in infected mice, six weeks following the infection. Indeed, and despite the reduced overall frequency of T_reg_ in *B. bronchiseptica* carriers, in vitro stimulation of splenocytes isolated from *B. bronchiseptica* carriers with heat-killed bacteria revealed the expansion of Bordetella-specific CD4^+^CD25^+^Foxp3^+^ T_reg_ within the polyclonal T_reg_ pool (Figure 3c). These results clearly showed distinct Bordetella-induced alterations to peripheral T_reg_. Based on these findings, we aimed at further characterizing the peripheral CD4^+^CD25^+^ T cell pool, which also contained CD4^+^CD25^+^Foxp3^+^ T_reg_, during *B. bronchiseptica* carriage, in an unbiased approach. To this end, we performed transcriptional profiling of CD4^+^CD25^+^ T cells isolated from the spleens of *B. bronchiseptica* infected and control mice on day 7 post infection, when peak bacterial loads were detected (acute infection); and day 42 post infection, when *B. bronchiseptica* specific CD4^+^ T cells, as well as T_reg_, were systemically detectable (carriage) (Figure 3d). Of note, we chose the late time point of day 42 post infection to address changes in the CD4^+^CD25^+^ T cell compartment that were mediated by carriage of *B. bronchiseptica*, but were not linked to the T cell activation mounted during peak pathogenic load, and during the subsequent generation of the specific adaptive immune response. Strikingly, whilst changes were only subtle during acute infection, transcriptional profiling showed that respiratory *B. bronchiseptica* carriage induced dramatic alterations in the expression profile of the peripheral polyclonal CD4^+^CD25^+^ T cell pool. Our analyses revealed 512 genes to be up-regulated and 80 genes to be down-regulated at least 2-fold in splenic CD4^+^CD25^+^ T cells isolated on day 42 post *B. bronchiseptica* infection, when compared to uninfected controls. In contrast, only 67 transcripts were up- and 4 transcripts were down-regulated on day 7 post *B. bronchiseptica* infection. Of note, CD4^+^CD25^−^ T cells that were analyzed in parallel only underwent minor changes at both time points (Supplementary Materials Figure S1a). Furthermore, the transcriptional changes observed in the CD4^+^CD25^−^ T cell compartment were largely independent from those regulated in the CD4^+^CD25^+^ T cell pool (Figure S1b). Table 1 summarizes the top 30 genes most up- and down-regulated exclusively in CD4^+^CD25^+^ T cells, but not in CD4^+^CD25^−^ T cells, isolated from *B. bronchiseptica* carriers, when compared to CD4^+^CD25^+^ T cells isolated from the respective uninfected controls. We detected transcriptional regulation of immunologically relevant proteins typically associated with the cell surface of T cells, such as CD38 (fold change 2.8) and Toll-like receptor (TLR) 4 (fold change 2.6). Additionally, IL-4 (fold change 2); the NFk-B inhibitor alpha (Nfkbia; fold change 2.1); and transcription factors such as the forkhead box P1 (Foxp1; fold change 2.8), interferon regulatory factor 2 (Irf2; fold change 4.7), and the RAR-related orphan receptor alpha (Rora; fold change 2.8) were up-regulated in CD4^+^CD25^+^ T cells during *B. bronchiseptica* carriage. Furthermore, the IL-2 receptor beta chain (Il2rb; fold change −2.2) and the transcription factor special AT-rich sequence binding protein 1 (Satb1; fold change −2.3) were down-regulated during carriage. To capture the overall significance of the broad transcriptional regulation in CD4^+^CD25^+^ T cells isolated from *B. bronchiseptica* carriers on a functional level, we performed gene ontology (GO)-term enrichment analysis on the list of transcripts regulated more than two-fold (Figure 4). Next to the immune-associated GO-terms “leukocyte differentiation” and “cytoplasmic pattern recognition receptor signaling pathway”, GO-terms of general cell biological processes, such as “transcription, DNA-templated” and “negative regulation of RNA metabolic processes” were significantly enriched in the transcripts regulated in CD4^+^CD25^+^ T cells isolated from *B. bronchiseptica* carriers. Nevertheless, the extensive regulation that exclusively occurred during *B. bronchiseptica* carriage, but not acute infection, suggested immune regulatory mechanisms rather than mere CD4^+^ T cell activation as a driving force. In line with this, typically T_reg_-associated transcripts such as neuropilin 1 (Nrp1; fold change 2.1) [37], were found to be transcriptionally up-regulated in the CD4^+^CD25^+^ T cell pool isolated from *B. bronchiseptica* carriers, as compared to uninfected controls. Importantly, comparing gene expression levels between CD4^+^CD25^+^ and CD4^+^CD25^−^ T cells revealed a clear T_reg_ signature for the CD4^+^CD25^+^ T cell pool, independent of *B. bronchiseptica* infection or carriage (Table 2). This T_reg_ signature was marked by transcriptional up-regulation of established T_reg_-associated factors, such as Gpr83 [38,39], CTLA4 [40], CD103 (Itgae) [41], the Zinc-finger protein Eos (Ikzf4) [42] and Foxp3 [43], all of which were among the 30 most intensely up-regulated transcripts, when comparing CD4^+^CD25^+^ and CD4^+^CD25^−^ T cells. Importantly, up-regulated expression of these T_reg_-associated factors also held true for CD4^+^CD25^+^ T cells isolated from *B. bronchiseptica* carriers.

Taken together, these analyses show that *B. bronchiseptica* carriage in the respiratory tract was associated with the expansion of Bordetella-specific CD4^+^CD25^+^Foxp3^+^ T_reg_, as well as with broad alterations in the transcriptional profile of the overall peripheral CD4^+^CD25^+^ T cell pool, which also pointed to the induction of T_reg_. In turn, these modulations to the peripheral T_reg_ pool displayed one possible mechanism by which leukocyte transfer from *B. bronchiseptica* carriers to acutely infected mice, hindered bacterial clearance in the recipient’s upper respiratory tract. Importantly, immune modulation through *B. bronchiseptica* carriage could also affect the responses to unrelated immunological stimuli in the lung or the periphery.

### 2.3. Respiratory B. bronchiseptica Carriage Does Not Alter the Humoral Immune Response to Vaccination

**Figure 5:**
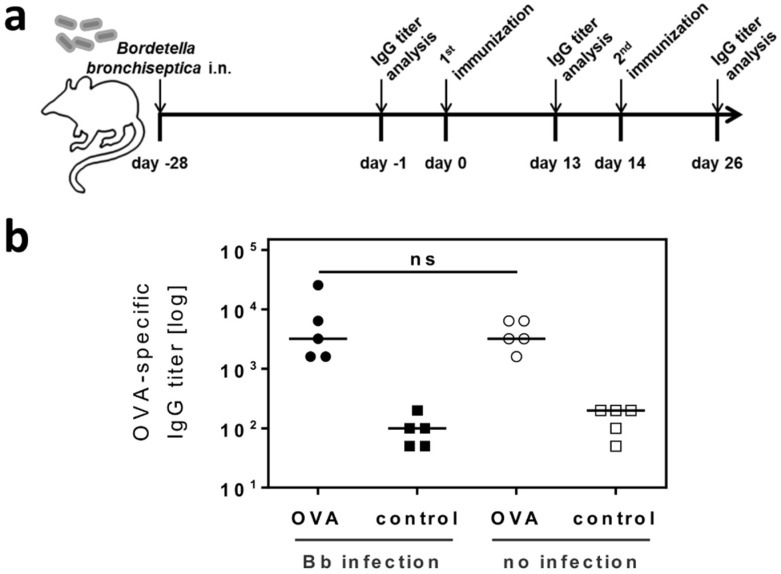
Respiratory *B. bronchiseptica* carriage does not alter the humoral immune response to vaccination. BALB/c mice were intranasally infected with 1 × 10^6^ CFU *B. bronchiseptica* or left uninfected for four weeks (day −28) before subcutaneous immunization with 10 µg OVA + Poly (I:C)/CpG, or with the adjuvants alone on days 0 and 14. OVA-specific serum IgG titers were determined for all animals at the indicated time points (days −1, 13 and 26). (**a**) Experimental setup. (**b**) OVA-specific total IgG titer of individual mice on day 26. Results are expressed as endpoint titers of individual mice and show the median/group. Groups were statistically analyzed using the one-way ANOVA, followed by Bonferroni´s post-test. ns = not significant (*p* > 0.05).

Possible influences of microbial carriage and microbiota on vaccination efficiency are currently being discussed [44,45,46,47]. Thus, we investigated whether immune modulation through respiratory *B. bronchiseptica* carriage would affect the immune response to vaccination with an unrelated antigen. To this end, *B. bronchiseptica* infected mice were first immunized subcutaneously with the model antigen ovalbumin (OVA) on day 28 post infection, followed by a second booster immunization with the same antigen 14 days later (Figure 5a). To quantify the antibody response mounted towards the model antigen, serum samples were collected from vaccinated *B. bronchiseptica* carriers, as well as control animals, which were analyzed for OVA-specific IgG titers (Figure 5b). These analyses revealed that *B. bronchiseptica* infected and uninfected animals responded equally well to vaccination. Therefore, respiratory carriage with *B. bronchiseptica* and the associated changes to the peripheral CD4^+^CD25^+^ T cell pool, did not alter humoral immunity to an unrelated antigen administered to the periphery.

### 2.4. Respiratory B. bronchiseptica Carriage Does Not Affect the Local Antiviral Immune Response Following Secondary Infection with Influenza A Virus

**Figure 6:**
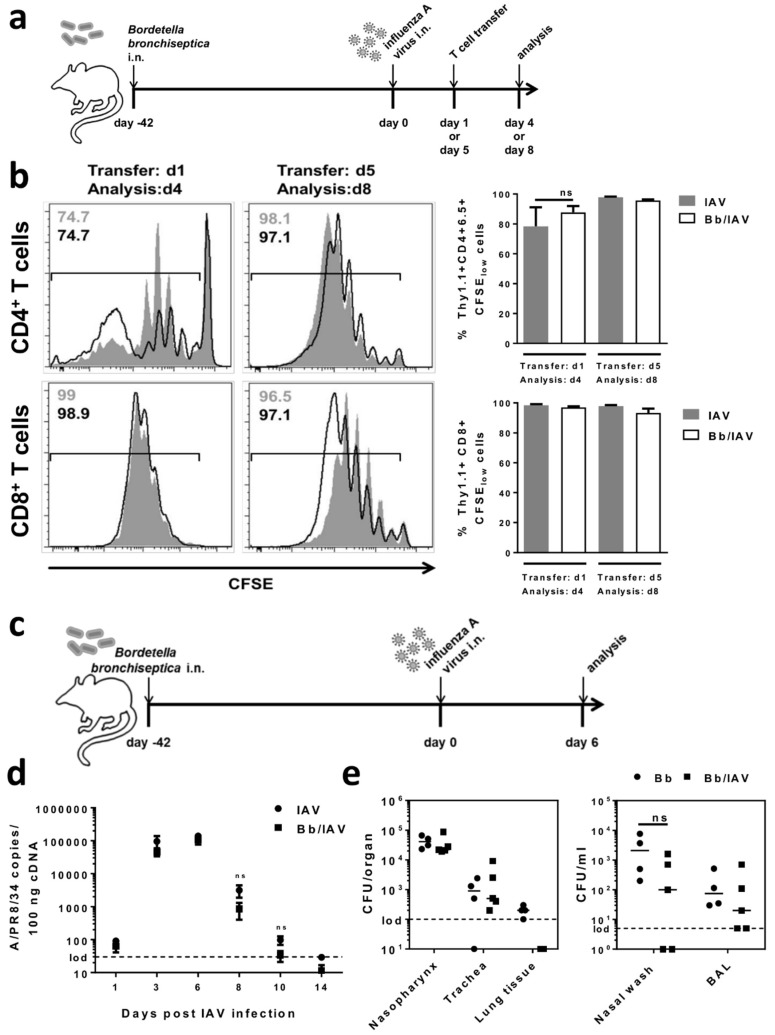
Respiratory *B. bronchiseptica* carriage does not affect the antiviral immune response towards secondary infection with influenza A virus. BALB/c mice were intranasally infected with 1 × 10^6^ CFU *B. bronchiseptica* (Bb) or left uninfected, followed by intranasal infection of all mice with 0.04 LD_50_ IAV PR8 on day 42. (**a**) Experimental setup. (**b**) Naive CD4^+^Thy1.1^+^ or CD8^+^Thy1.1^+^ T lymphocytes isolated from spleens and cervical lymph nodes of TCR-HA mice (CD4^+^ T cells) or CL4 mice (CD8^+^ T cells) were labelled with carboxyfluorescein diacetate succinimidyl ester (CFSE). Cells were adoptively transferred into infected mice on day 1 or day 5 post IAV infection. On day 3, following adoptive transfer, lymphocytes were recovered from the bronchial lymph nodes and proliferation was determined by the analysis of CFSE dilution. Histogram plots show overlays of adoptively transferred cells in the two experimental groups: black lines for the IAV-only infected groups and grey shades for the IAV infected *B. bronchiseptica* carriers. The respective percentage of HA-specific T cells that underwent proliferation (CFSE_low_) is indicated. Data were compiled from two independent experiments (*n =* 4–6 mice/group). Bar graphs indicate the mean ± SEM/group. Groups were compared using the two-tailed Mann-Whitney test. (**c**) Experimental setup. (**d**) At the indicated time points, RNA was extracted from the lung tissue and IAV genome copies were quantified by qRT-PCR. Data are shown as mean ± SEM of *n =* 4–5 mice per group and time point. The dashed line indicates the limit of detection. (**e**) Mice were intranasally inoculated with 1.5 × 10^6^ CFU of *B. bronchiseptica* (Bb) and with IAV or PBS, according to the experimental setup shown in (**c**). On day 6, following IAV infection, *B. bronchiseptica* CFU were determined in homogenized lung and tracheal tissue, the nasopharynx, as well as in bronchoalveolar lavage (BAL) and nasal wash. Data show individual mice and the median/group. Dashed lines indicate the limit of detection. Groups were compared by unpaired, two-tailed Mann-Whitney test. ns = not significant (*p* > 0.05).

In addition to assessing the effects of adoptively transferred immune cells of *B. bronchiseptica* carriers on bacterial clearance in an acute *B. bronchiseptica* challenge, we asked whether carriage would also impinge on cellular adaptive immunity to a secondary respiratory tract infection with an unrelated pathogen. To experimentally address this issue, we employed an antigen-specific adoptive T cell transfer system, in combination with a respiratory influenza A virus (IAV) infection (Figure 6a). Respiratory *B. bronchiseptica* carriers were infected with IAV. To enable the analysis of antigen-specific T cell proliferation in this model, the mice received adoptive transfer of either CD4^+^ or CD8^+^ T cells, specific for the IAV hemagglutinin (HA) on day 1 or day 5 following IAV infection. Both the IAV-specific CD4^+^ and CD8^+^ T cells that were recovered from the draining lymph nodes of IAV only infected recipient mice and of IAV infected *B. bronchiseptica* carriers, had proliferated equally well in response to viral antigen recognition, as shown in Figure 6b. Of note, the dissemination of proliferated HA-specific CD4^+^ and CD8^+^ T cells to the periphery was unchanged (Supplementary Materials Figure S2). These observations indicated that *B. bronchiseptica* carriage in the respiratory tract did not negatively affect antigen-presentation and the subsequent induction of specific T cell responses to a secondary viral pathogen in the lung. To assess possible effects of *B. bronchiseptica* carriage on the anti-IAV immune response that were independent of the unchanged T cell response, e.g., on innate antiviral defense, we analyzed viral clearance in *B. bronchiseptica* carriers infected with IAV (Figure 6c). The viral load of the lung was quantified at different time points over the course of the infection (Figure 6d). However, respiratory *B. bronchiseptica* carriage neither affected early viral replication nor clearance from the lung, suggesting equally efficient anti-viral mechanisms to be present in *B. bronchiseptica* carriers. Importantly, respiratory IAV infection triggers a full anti-viral immune response even at sublethal doses and causes substantial inflammation in the respiratory tract, involving a variety of different immune cell types, as well as inflammatory and anti-inflammatory mechanisms [48,49]. Therefore, effects of the IAV infection on *B. bronchiseptica* carriage and distribution within the respiratory tract were well conceivable. Thus, we quantified bacterial carriage in IAV infected *B. bronchiseptica* carriers at the peak of viral replication (day 6 post IAV infection), and there was a trend towards improved bacterial clearance in the lung and nose of IAV infected *B. bronchiseptica* carriers (Figure 6e). However, this did not reach statistical significance, demonstrating that secondary IAV infection, neither significantly boosted antibacterial immune mechanisms nor supported the outgrowth of *B. bronchiseptica* in the upper or lower respiratory tract of carriers. Taken together, these analyses showed that respiratory *B. bronchiseptica* carriage and the associated changes to the immune system do not affect immunity to secondary IAV infection in the lung.

### 2.5. Respiratory B. bronchiseptica Carriage Alters Immunity to Systemic L. monocytogenes Infection

**Figure 7:**
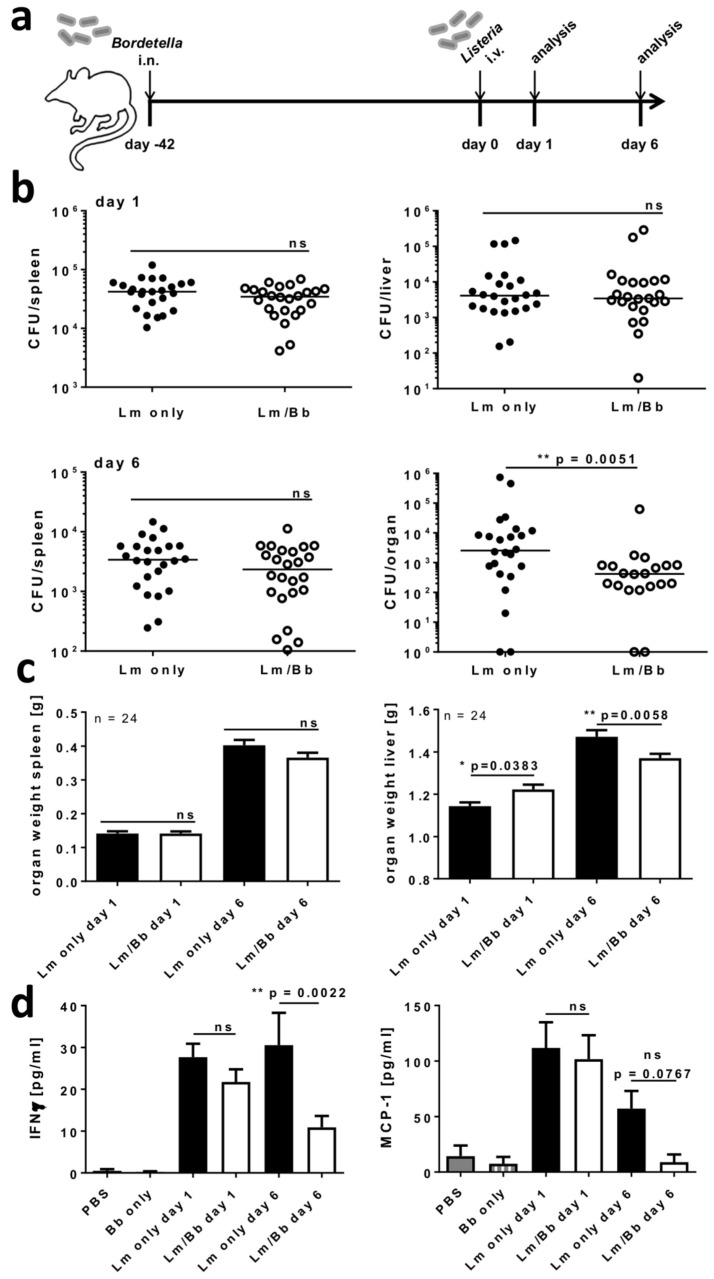
Respiratory *B. bronchiseptica* carriage alters immunity to systemic *L. monocytogenes* infection. BALB/c mice were intranasally infected with 1 × 10^6^ CFU of *B. bronchiseptica* (Bb) or left uninfected, followed by secondary systemic *L. monocytogenes* (Lm) infection. (**a**) Experimental setup. On day 1 or day 6 post Lm infection the bacterial burden in the spleen and the liver (**b**), the organ weight (**c**), and the levels of IFN-γ and MCP-1 in the serum (**d**) of these mice were assessed. In (**b**) values for single mice and the group median, and in (**c**)/(**d**) group means ± SEM of 24 mice/group and 9–16 mice/group, respectively, are shown. Data are compiled from three independent experiments, and groups were compared by unpaired, two-tailed Mann-Whitney test in (b) and one-way ANOVA, followed by Bonferroni’s post-test in (**c**) and (**d**). ns = not significant (*p* > 0.05).

IAV, like *B. bronchiseptica*, is a respiratory pathogen, and thus, we further assessed the effect of *B. bronchiseptica* carriage on a secondary systemic infection by *L. monocytogenes*. To this end, mice were first intranasally infected with *B. bronchiseptica,* followed by intravenous infection with a sublethal dose of *L. monocytogenes* six weeks later (Figure 7a). We assessed the organ dissemination of *L. monocytogenes* by quantifying bacterial loads in the spleen and the liver, on days 1 and 6 post infection (Figure 7b). Strikingly, while *L. monocytogenes* loads were unchanged in the spleen at both time points and in the liver early post infection, we detected a significantly reduced *L. monocytogenes* burden in the livers of *B. bronchiseptica* carriers, compared to only *L. monocytogenes* infected mice six days after the secondary infection. These results suggested a significant benefit from respiratory *B. bronchiseptica* carriage, for the clearance of *L. monocytogenes* in the liver. Next to the *L. monocytogenes* bacterial burden, we assessed the organ weight as a measure for inflammation and tissue damage [50] (Figure 7c). While no differences were detected for the spleens, the liver weight was significantly reduced in *L. monocytogenes* infected *B. bronchiseptica* carriers on day 6 following secondary infection, compared to only *L. monocytogenes* infected mice. This observation was well in line with the significantly reduced bacterial burden in the liver at this time point. Systemic *L. monocytogenes* infection is associated with a substantial inflammatory response [51]. Thus, in a next step, we assessed the pro-inflammatory response and evaluated if it correlated with the effects of *B. bronchiseptica* carriage on *L. monocytogenes* loads in the liver. To this end, we assessed the levels of interferon (IFN)-γ and monocyte chemotactic protein 1 (MCP-1) in the serum of *L. monocytogenes* infected mice (Figure 7d). We detected a strong induction of both mediators in the serum of *L. monocytogenes* infected naïve mice, as well as *B. bronchiseptica* carriers, on day 1 following *L. monocytogenes* infection. Strikingly, both IFN-γ and MCP-1 were strongly reduced in *L. monocytogenes* infected *B. bronchiseptica* carriers, compared to only *L. monocytogenes* infected mice, by day 6 following infection. These results were well in line with the reduced pathogen burden in the liver, detected in *L. monocytogenes* infected *B. bronchiseptica* carriers at this time point. Ultimately, these data indicated that chronic respiratory *B. bronchiseptica* carriage, significantly affects peripheral immunity towards an unrelated secondary bacterial pathogen.

## 3. Discussion

Immune reactions are versatile, well-coordinated processes involving a number of different players. Next to immune activation, tight regulatory mechanisms are in place to protect the host from immune mediated pathology. Such regulation can, however, also result in pathogen immune evasion and promotion of persistence. Currently, increasing attention is being directed to the interactions between responses to different simultaneous or sequential immunologically relevant stimuli in single hosts. Furthermore, next to the gut and skin, the lower respiratory tract is now being acknowledged as a niche for colonization by harmless and potentially harmful microorganisms [1,2]. The questions arising are, what regulatory mechanisms are potentially induced through respiratory pathogen carriage and how these affect the response to secondary immunological triggers. Our study comprehensively addresses these questions by in vivo utilizing *B. bronchiseptica* as a mouse model, for carriage with a potentially pathogenic microorganism. Of note, while *B. bronchiseptica* infection has frequently served as a mouse model for studies on the closely related major human pathogen *B. pertussis* in the past [21], our study employed *B. bronchiseptica* primarily as a robust model for respiratory microbial carriage in the mouse.

We clearly show immune modulatory effects resulting from respiratory *B. bronchiseptica* carriage. This was demonstrated by the adoptive transfer of leukocytes isolated from carriers to acutely *B. bronchiseptica* infected mice, which resulted in significantly increased bacterial numbers in the recipient’s upper respiratory tract. The induction of immunosuppressive cells such as T_reg_ during *B. bronchiseptica* carriage, represents one possible mechanism underlying this observation. Interestingly, T_reg_ induction has been described for *B. pertussis* infected mice and has been implicated and discussed, however, it has not been addressed experimentally for *B. bronchiseptica* infection [35,36,52]. We now show the induction of *Bordetella*-specific T_reg_ following respiratory infection. Furthermore, our analyses of the peripheral CD4^+^CD25^+^ T cell pool revealed exceptionally broad transcriptional changes, induced by respiratory *B. bronchiseptica* carriage. These broad changes together with the detected imbalance in favor of up-regulated over down-regulated transcripts revealed transcriptional activation in CD4^+^CD25^+^ T cells of chronic *B. bronchiseptica* carriers that was probably associated with some gain or change in function rather than mere functional suppression. However, the most intensely up-regulated transcripts in CD4^+^CD25^+^ T cells during chronic carriage (listed in Table 1) do not clearly associate with any apparent annotated function in the defense against bacterial pathogens or the suppression of immune responses. Instead, GO-term analysis of all the genes differentially expressed in CD4^+^CD25^+^ T cells isolated from *B. bronchiseptica* carriers revealed that mainly transcription- and RNA-metabolism-associated genes are regulated. Nevertheless, up-regulated transcripts did include genes known to be associated to immune responses mounted towards *B. bronchiseptica* or *B. pertussis*. TLR4, which was up-regulated 2.6-fold in CD4^+^CD25^+^ T cells from *B. bronchiseptica* carriers, compared to uninfected controls, has been described to play a role in very early infection with *B. bronchiseptica* [53] and to affect transmission [54]. Nevertheless, there are also implications for its up-regulation by CD4^+^CD25^+^ T cells, as distinct TLR4-mediated responses of T cells have been reported [55]. Il-4 (2.0-fold up-regulated in CD4^+^CD25^+^ T cells from *B. bronchiseptica* carriers, compared to uninfected controls), has been suggested to display an important regulator of the recruitment of inflammatory cells in the context of *B. pertussis* challenge, following vaccination [56]. Importantly, the detected transcriptional changes in the CD4^+^CD25^+^ T cell pool of *B. bronchiseptica* carriers were predominantly detected six, but not one week, following infection, and thereby pointed to carriage-associated mechanisms that were most likely of a regulatory manner. Indeed, this conclusion is supported by the finding that several T_reg_-associated transcripts were differentially expressed in CD4^+^CD25^+^ T cells during respiratory carriage, compared to CD4^+^CD25^+^ T cells isolated from controls. Here, Nrp1 stands out, in as much as it is a murine T_reg_-marker not expressed in activated conventional T cells [37]. Nrp1 promotes long-lasting interactions between T_reg_ and immature DC, and functions as receptor for immune suppressive TGF-ß [57]. Moreover, up-regulation of Nfkbia, an inhibitor of NFκB, suggests reduced responsiveness to T cell stimulation, which typically involves activation of the NFκB pathway [58]. Importantly, CD4^+^CD25^+^ T cells generally, and also during *B. bronchiseptica* carriage, revealed a clear transcriptional T_reg_ signature when compared to the CD4^+^CD25^−^ T cell pool isolated at the same time. Here, well established T_reg_-specific factors, such as Gpr83 [38,39], CTLA4 [40], CD103 [41], the Zinc-finger protein Eos (Ikzf4) [42] and Foxp3 [43] were among the 30 most intensely up-regulated transcripts, pointing to the strong presence of T_reg_ in the CD4^+^CD25^+^ T cell pool, also during respiratory *B. bronchiseptica* carriage. Functionally, it has been shown that CD4^+^CD25^+^Foxp3^+^ T_reg_ not only act on adaptive lymphocytes, but also affect innate immune cells. Here, T_reg_ have the potential to induce the alternative activation of monocytes/macrophages [59]. In line with this, persistent colonization with *B. bronchiseptica* has been suggested to rely on the differential modulation of both macrophage and DC function, in turn leading to an altered adaptive immune response [31]. Therefore, the T_reg_ signature detected in CD4^+^CD25^+^ T cells isolated from *B. bronchiseptica* carriers as compared to the respective CD4^+^CD25^−^ T cell population, together with the detection of *B. bronchiseptica*-specific T_reg_ during carriage, suggest T_reg_-associated mechanisms of *B. bronchiseptica* carriage. Additionally, it makes a transfer of suppressive T_reg_ together with the adoptively transferred leukocytes from *B. bronchiseptica* carriers likely and is a possible mechanism underlying the increased bacterial load in acutely infected recipients. Generally, the regulatory mechanisms that act in the respiratory tract, next to T_reg_, also include but are not restricted to alternatively activated (M2) macrophages and myeloid derived suppressor cells (MDSC) [3,4,7,8]. Therefore, at this point, it remains unclear whether the effects we observed for the adoptive transfer of leukocytes from *B. bronchiseptica* carriers into acutely infected hosts were linked to the accumulation of T_reg_ specific for *B. bronchiseptica*; or to overall changes in the CD4^+^CD25^+^ T cell pool; or to independent mechanisms, such as the transfer of likewise induced MDSC. Future studies will have to address these details and will have to clarify the mechanisms interfering with bacterial clearance in the upper respiratory tract, following the adoptive transfer of leukocytes isolated from *B. bronchiseptica* carriers. Nevertheless, our results clearly show the regulatory potential of respiratory *B. bronchiseptica* carriage.

With the clear immune modulatory effects observed, we hypothesized that *B. bronchiseptica* carriage had the potential to also affect responses to unrelated immunological stimuli, such as vaccination and infection. Of note, possible influences of microbial carriage on vaccination efficiencies are currently being discussed [44,45,46,47,60]. However, we found that *B. bronchiseptica* carriers mounted a full humoral response to subcutaneous vaccination with a model antigen. This response was unchanged when compared to naïve control mice, suggesting that under these experimental conditions, respiratory colonization with a potentially pathogenic microorganism neither displayed beneficial nor adverse effects on vaccination efficacy. In contrast to vaccination, infections are typically accompanied by pathogen replication and at least some extent of host cell damage, which together lead to stronger inflammation and immune stimulation, despite the use of adjuvants in vaccination. For IAV, strong anti-viral responses and collateral immune pathology are hallmark features of the infection, and in turn there is a particular need for regulation to protect the host from immune mediated damage [48,61]. Therefore, we hypothesized that adaptive anti-IAV responses might be exceptionally “susceptible” to the regulatory mechanisms induced by *B. bronchiseptica* carriage. Of note, *B. bronchiseptica* pulsed macrophages have previously been shown to inhibit antigen-specific CD4^+^ T cell proliferation in vitro [31]. However, we detected unchanged IAV-specific T cell proliferation and dissemination in *B. bronchiseptica* carriers, compared to naïve mice following IAV infection. Therefore, presumably antigen-presentation following the viral infection was fully functional, despite the effects of *B. bronchiseptica* on macrophages and DC, which have previously been described in References [30,33,36]. Importantly, viral clearance from the respiratory tract was equally efficient in *B. bronchiseptica* carriers, pointing at likewise intact innate responses. This observation was somewhat in contrast to the findings of a human study, describing asymptomatic neonatal airway colonization with different respiratory pathogens to increase the risk of pneumonia and bronchiolitis in early life [62]. Moreover, pre-infection with *B. bronchiseptica* has been shown to favor colonization with *Haemophilus parasuis,* and simultaneous acute infection with *B. bronchiseptica* and IAV has been shown to lead to increased bacterial titers in swine [63,64]. In our model, we focused on respiratory carriage with *B. bronchiseptica*, which neither affected clearance of the viral respiratory pathogen nor the underlying respiratory bacterial carriage. Such effects most likely do not represent general mechanisms, but depend on the specific microorganisms, the phase of the infection, as well as on the age and underlying conditions of the host. For example, neonates, as were studied in Reference [58], harbor a still developing and thereby unique immune system [65], where microbial carriage most likely has different effects than later in life. In addition, distinct microbiological features of the airway microbiome have been associated with specific respiratory diseases [66], underlining the complexity of respiratory immune regulation with respect to microbial carriage.

Despite the fact that respiratory IAV infection went unchanged, we explored whether respiratory carriage with *B. bronchiseptica* affected responses against a systemic bacterial infection. Of note, only recently asymptomatic colonization with *Moraxella catharralis*, *Haemophilus influenzae,* and/or *Streptococcus pneumoniae* in the hypopharynx, was shown to be associated with low-grade systemic inflammation in new-born children [18]. Surprisingly, we found *B. bronchiseptica* carriage to display a significant benefit for the host, regarding the control of systemic *L. monocytogenes* infection. Next to decreased bacterial numbers in the liver, we further detected reduced levels of MCP-1 and IFN-γ in the serum of *L. monocytogenes* infected *B. bronchiseptica* carriers. In line with this, the *B. bronchiseptica* T3SS has been shown to inhibit IFN-γ production via IL-10 to promote persistent carriage [35]. *B. bronchiseptica* mediated inhibition of IFN-γ production, could in turn possibly have led to the reduced bacterial burden in the liver, as a consequence of reduced inflammation and immune pathology. Such a mechanistic link is strongly supported by the observation that excessive IFN-γ production following *L. monocytogenes* infection, exacerbates pathology and impairs anti-bacterial defense mechanisms [67]. Ultimately, our study clearly shows the potential of respiratory carriage with a pathogenic microorganism, to significantly alter systemic responses to a secondary bacterial infection.

Taken together, our results show clear and significant effects of respiratory *B. bronchiseptica* carriage on the immune system. Considering the results of previous studies, these immune modulatory processes are most likely aimed at supporting microbial persistence in the host [34,35,36]. We have now for the first time comprehensively addressed their significance, for secondary immune responses in vivo, employing *B. bronchiseptica* as a model for respiratory pathogen carriage. We did not observe general, but highly differential effects, depending on the considered compartment and the nature of the secondary stimulus. Interestingly, the effects we described can be beneficial for the pathogen on the one hand, as observed in the adoptive transfer experiments, or beneficial for the host, as demonstrated by the improved containment of *L. monocytogenes* in the livers of *B. bronchiseptica* carriers. Therefore, our results demonstrated the complexity of the versatile local and systemic immune regulation, induced by respiratory carriage with a pathogenic microorganism. Furthermore, our study provided a valuable basis for future investigations, which will address the decisive factors and elucidate specific mechanisms of such interactions, that will be essential for prophylactic and therapeutic considerations.

## 4. Materials and Methods

### 4.1. Mice

BALB/c mice were obtained from Harlan (Borchen, Germany). Clone 4 (CL4) mice expressing a major histocompatibility complex (MHC)-I–restricted HA-specific T cell receptor, and TCR-HA mice expressing a MHC-II-restricted T cell receptor, have been described previously in References [68,69]. Transgenic T cell receptor bearing mice were used on the Thy1.1 congenic background to allow for tracking of HA-specific T cells, after adoptive transfer into BALB/c recipient mice of the Thy1.2 congenic background. Transgenic mice were bred under specific pathogen-free conditions, at the Helmholtz Centre for Infection Research (Braunschweig, Germany). Animal experiments were performed according to national and institutional guidelines, under the project identification codes 11/0425, 10/0108, and 10/0256 (Nds. Landesamt für Verbraucherschutz und Lebensmittelsicherheit).

### 4.2. Bacterial and Viral Pathogens

*B. bronchispetica* wild type strain BB7865 [70] was obtained from the Culture Collection of the University of Gothenburg, Sweden. Bacteria were prepared as described in Reference [20]. Briefly, *B. bronchiseptica* was grown for 24 h, on Bordet-Gengou agar plates (supplemented with 10% sheep blood and 100 µg/mL streptomycin). Colonies were suspended in PBS, the OD_600nm_ was determined, and bacterial inocula were prepared by dilution. The dose/mouse was retrospectively validated by plating serial dilutions of the inoculum.

Madin-Darby canine kidney (MDCK) cell-derived IAV PR8/A/34 (H1N1), was obtained as described in Reference [71] and diluted in PBS to the appropriate concentration before infection.

*L. monocytogenes* strain EGD was grown in Brain Heart Infusion (BHI). For mouse infections, bacteria were collected in mid-log phase, centrifuged, and resuspended in PBS to the desired concentration. The dose/mouse was retrospectively validated by plating serial dilutions of the inoculum on BHI agar plates.

### 4.3. Bacterial and Viral Infections

For intranasal *B. bronchiseptica* infection, mice were lightly anaesthetized by isoflurane inhalation, and 20 µL (10 µL per nostril) of the bacterial inoculum (~ 0.5–1 × 10^6^ CFU) were administered. For intranasal IAV infection, mice were infected with 10^0.9^ TCID_50_ of the virus in 25 µL PBS under ketamine/xylazine anesthesia. Holding the animals upright, the viral or bacterial inoculum was delivered to the nostrils to be taken up by the mouse upon breathing.

For systemic *L. monocytogenes* infection, 2 × 10^3^ CFU/mouse in sterile PBS were intravenously injected into the tail vein.

### 4.4. Quantification of Pathogen Loads Following Infection

For the quantification of *B. bronchiseptica* CFU, bronchoalveolar lavage (BAL) was obtained by flushing the lungs with 1 mL PBS through the trachea. Nasal wash fluid was obtained by flushing the nasopharynx with 200 µL PBS. Lungs and tracheas were aseptically removed and homogenized separately in PBS (Polytron^®^ PT 1300D, Kinematica, Lucerne, Switzerland). Serial dilutions of airway fluids and tissue homogenates were plated onto LB agar plates (supplemented with streptomycin), incubated overnight, and CFU counts were calculated.

For the quantification of the lung viral load following IAV infection, lungs were homogenized in TriFastFL reagent (PeqLab now VWR Life Science Competence Center, Erlangen, Germany). RNA was extracted by addition of 1-Br-3-Cl-Propane (Merck, Darmstadt, Germany), purified by precipitation and treated with DNase (Ambion, Thermo Fisher Scientific, Waltham, MA, USA), prior to reverse transcription to cDNA (M-MLV Reverse Transcriptase; Invitrogen, Thermo Fisher Scientific, Waltham, MA, USA). Quantitative real-time PCR of 100 ng cDNA samples, was conducted using the LightCycler^®^ 480 SYBR Green I Master Kit (Roche, Basel, Switzerland) and primers specific for the IAV nucleoprotein (NP). Quantitative real-time PCR of samples containing known numbers of a plasmid carrying the NP sequence (pVI-PmH5-PR8-NP; provided by G. Sutter, Munich, Germany) was performed to obtain a CT-value/NP copy number standard, which was used to quantify NP copy numbers in the samples. NP primers were GAGGGGTGAGAATGGACGAAAAAC (5′-NP) and CAGGCAGGCAGGCAGGACTT (3′-NP) and were used in a final concentration of 500 nmol/L. Quantitative real-time PCR was performed using a LightCycler^®^ instrument.

For the quantification of *L. monocytogenes* bacterial counts, spleens and livers (without gall bladder) were harvested from infected mice, weighed, and homogenized (Polytron^®^ PT 1300D, Kinematica, Lucerne, Switzerland) in PBS containing 0.2% IGEPAL CA-630. Organ homogenates were plated on BHI agar plates, and CFU were counted after 24 h of incubation.

### 4.5. Restimulation of Splenocytes with Heat-Killed B. bronchiseptica

*B. bronchiseptica* was cultured on Bordet-Gengou agar plates for 24 h at 37 °C, and resuspended and diluted to the desired concentration in sterile PBS. Bacteria were heat-inactivated for 30 min at 55–56 °C. Splenocytes were isolated from groups of three mice per group, were pooled, and re-stimulated in vitro, with or without heat killed bacteria in a *B. bronchiseptica*-to-cell ratio of 10:1, for 4 days (2 × 10^6^ cells/well). As a positive control for proliferation, lymphocytes were stimulated with Concavalin A (5 µg/mL; Sigma-Aldrich, Schnelldorf, Germany).

### 4.6. Flow Cytometry

For lymphocyte isolation, the spleens, cervical lymph nodes and bronchial lymph nodes were rinsed with PBS and meshed through a 100 µm cell strainer followed by centrifugation and the lysis of red blood cells through osmotic shock. Antibody staining of up to 10^6^ cells was performed in 96-well plates. Cells were washed in FACS (fluorescence activated cell sorting) buffer (PBS, 2% [*v*/*v*] fetal bovine serum, 2 mM EDTA) and incubated with the indicated antibodies (fluorochrome-conjugated) diluted in FACS buffer for 10 min at 4 °C in the dark. Antibody concentrations were separately titrated for each antibody, fluorophore and lot. Following surface staining, cells were washed and immediately analyzed or alternatively fixed in 100 µL/sample 1% (*w*/*v*) paraformaldehyde in PBS for 20 min at room temperature. For the intracellular staining of Foxp3 following surface staining, the Foxp3 staining buffer set (eBioscience, Thermo Fisher Scientific, Waltham, MA, USA) was used according to the manufacturer’s recommendations. Acquisition of flow cytometry samples was performed on a FACSCanto (BD, Franklin Lakes, NJ, USA), and data were analyzed with the FlowJo software (Tree Star, Becton, Dickinson & Company, Franklin Lakes, NJ, USA). A monoclonal antibody specific to mouse CD90.1 (OX-7) was obtained from BioLegend (San Diego, CA, USA). Antibodies specific to mouse CD4 (RM4-5), CD8 (53-6.7) and Foxp3 (FJK-16s) were obtained from eBioscience (Thermo Fisher Scientific, Waltham, MA, USA). Antibodies specific for mouse CD25 (PC61 and 7D4) were obtained from BD Pharmingen (Franklin Lakes, NJ, USA). In addition, HA-specific CD4^+^ T cells were identified using α-6.5 (α-TCR-HA; Clone: 14.3d) antibody, purified from hybridoma supernatant by affinity chromatography.

### 4.7. Adoptive Transfer of Leukocytes Isolated from B. bronchiseptica Carriers

For leukocyte isolation; spleens, cervical lymph nodes, and bronchial lymph nodes were rinsed with PBS and meshed through a 100 µm cell strainer, followed by centrifugation and lysis of red blood cells through osmotic shock. For adoptive transfer, leukocytes were labelled with a biotinylated CD45R/B22 antibody (RA3-6B2), and B-cell depleted using anti-biotin Micro-Beads (Miltenyi Biotec, Bergisch Gladbach, Germany) and an AutoMACS device, according to the manufacturer’s instructions. Recipient mice received 8 × 10^6^ cells in PBS intravenously. 

### 4.8. Microarray Analyses of Sorted CD4^+^CD25^+^ T Cells

Gene expression arrays were performed in the microarray facility of the Helmholtz Centre for Infection Research Braunschweig. Splenocytes from both infected and control mice were isolated and pooled (*n =* 6 per group) on day 7 or 42, post infection. CD4^+^CD25^+^ cells were flow cytometrically sorted on a BD FACSAria II instrument. Total RNA of sorted CD4^+^CD25^+^ cells was isolated using the Rneasy Mini Kit (Quiagen, Hilden, Germany), including the enzymatic digestion of DNA (Rnase-free Dnase Set, Quiagen), according to the manufacturer’s recommendations. RNA was concentrated by ethanol precipitation following elution, and RNA integrity was tested using an Agilent 2100 Bioanalyzer (Agilent Technologies, Santa Clara, CA, USA). Equal amounts of RNA were amplified using a T7dT23 primer (Metabion, Steinkirchen, Germany) and SuperScript II reverse transcriptase (Invitrogen). Second-strand cDNA synthesis was performed using DNA-polymerase I (*Escherichia coli* DNA ligase; Invitrogen). Double-stranded cDNA was amplified using the Promega P1300 RiboMax Kit, for T7 amplification (Promega, Madison, WI, USA). Resulting cRNA was amplified for first-strand synthesis of cDNA using random hexamer primers (Pharmacia, GE Healthcare, Chicago, IL, USA). Degradation of cRNA was performed through RNase H treatment, and second-strand synthesis was performed as described above, using T7dT23 primers. A second in vitro transcription was performed using the GeneChip expression 3’-Amplification Reagent Kit (Affymetrix, Santa Clara, CA, USA). Recommendations of the Affymetrix protocol, were followed for hybridization with the GeneChip Mouse Genome 430 2.0 Array (Affymetrix, Santa Clara, CA, USA), staining and scanning. Microarray data were analyzed using the GeneSpring GX 10.0 software (Agilent Technologies, Santa Clara, CA, USA), applying the RMA algorithm, including a quantile normalization. Gene ontology enrichment analyses were performed using the ClueGO Cytoscape plug-in software, as outlined in Reference [72]. Microarray data were deposited in the Gene Expression Omnibus (GEO), under the reference ID GSE116913.

### 4.9. Immunization with the Model Antigen OVA

Mice were subcutaneously (s.c.) immunized on days 0 and 14; either with 10 µg EndoGrade OVA (>98% purity) (Hyglos, Bernried, Germany), in addition to 50 µg Poly (I:C) (Invivogen, San Diego, CA, USA) and 50 µg CpG (Eurofins MWG Operon, Ebersberg, Germany) in a total volume of 50 µL PBS per animal, or with the adjuvants alone as control.

### 4.10. Detection of OVA-Specific Serum IgG

For the preparation of serum samples, 75 µL of blood were collected from the retro-orbital sinus, and samples were incubated for 45 min at 37 °C, followed by 45 min incubation at 4 °C, and subsequent centrifugation (10 min at 420× *g*). Sera were assayed for the presence of OVA-specific IgG by enzyme-linked immunosorbent assay (ELISA), using 96-well Nunc-Immuno MaxiSorp plates (Nunc, Thermo Fisher Scientific, Waltham, MA, USA) coated with 2 µg/mL OVA protein, in 0.05 M carbonate buffer (pH 9.6). After overnight coating at 4 °C, the plates were washed with PBS supplemented with 0.1% Tween 20, and blocked with 3% bovine serum albumin (BSA) in PBS for 1 h at 37 °C. Serial 2-fold dilutions of sera in 3% BSA/PBS were added and the plates were incubated for 2 h at 37 °C. After washing, antibody binding was detected using biotin-conjugated goat anti-mouse IgG (Sigma-Aldrich, Schnelldorf, Germany) (1 h, 37 °C) and Streptavidin-HRPO (BD Biosciences, Franklin Lakes, NJ, USA) (30 min, 37 °C). ABTS (2,2′-azino-bis (3-ethylbenzothioazoline-6-sulfonic acid) diammonium salt) in 0.1 M citrate-phosphate buffer (pH 4.35) containing 0.03% H_2_O_2_ was added to each well, and the absorbance at 405 nm was recorded after 30 min of incubation. Endpoint titers were expressed as the reciprocal value of the last serum dilution, which yielded an absorbance two times above the values of negative controls.

### 4.11. Adoptive Transfer of IAV-Specific CD4^+^ and CD8^+^ T Cells

CD4^+^ and CD8^+^ T cells were isolated from the spleen and cervical lymph nodes of TCR-HA or CL4 mice, and purified by negative selection, using the CD4^+^ and CD8^+^ T cell isolation kit and an AutoMACS instrument (Miltenyi Biotec, Bergisch Gladbach, Germany), respectively. Purified T cells were labeled with 2.5 µM carboxyfluorescein diacetate succinimidyl ester (CFSE; Invitrogen, Thermo Fisher Scientific, Waltham, MA, USA) and i.v. injected into the tail veins (1.5–1.8 × 10^6^ HA-specific CD4^+^ T cells, 5–7 × 10^6^ HA-specific CD8^+^ T cells) of recipient mice. Mice were sacrificed 3 days after T cell transfer and T cell proliferation in the bronchial lymph nodes (BLN), and spleen was assessed by flow cytometry.

### 4.12. Quantification of IFN-γ and MCP-1 in Serum

Serum was prepared as described above. Cytokine levels of IFN-γ and MCP-1 in serum samples were quantified using a BD™ Cytometric Bead Array (CBA) from BD Biosciences (Franklin Lakes, NJ, USA), following the manufacturer’s instructions.

### 4.13. Statistical Analyses

Groups were compared by the indicated statistical test, using the Graph Pad Prism software (Graph Pad Software, La Jolla, CA, USA). *p* values < 0.05 were considered to indicate statistical significance.

## Figures and Tables

**Table 1:** Top-regulated genes in CD4^+^CD25^+^ T cells isolated from *B. bronchiseptica* carriers.

Probe Set ID	Gene Symbol	Day 7	Day 42
1438082_at	Tmem206	1.1	**7.8**
1437641_at	Rprd2	−1.0	**6.7**
1433575_at	Sox4	1.6	**6.4**
1437668_at	Ccrl1	1.1	**5.4**
1436981_a_at	Ywhaz	−1.0	**5.3**
1452247_at	Fxr1	−1.5	**5.3**
1421144_at	Rpgrip1	1.5	**4.9**
1418265_s_at	Irf2	1.2	**4.7**
1431037_a_at	Elavl1	1.2	**4.5**
1438908_at	Map3k12	1.1	**4.4**
1436567_a_at	Ndufa7	1.3	**4.4**
1419913_at	Strap	−1.1	**4.3**
1419918_at	Tmed7	1.2	**4.3**
1418199_at	Hemgn	1.4	**4.2**
1426358_at	Taok1	1.3	**4.2**
1448011_at	Vps13c	−1.0	**4.1**
1419924_at	Fnip1	1.1	**4.0**
1422307_at	Sppl3	−1.1	**4.0**
1420175_at	Tax1bp1	−1.1	**3.9**
1422185_a_at	Cyb5r3	−1.1	**3.9**
1454169_a_at	Epsti1	1.0	**3.9**
1438216_at	Rreb1	−1.1	**3.8**
1431645_a_at	Gdi2	−1.2	**3.8**
1429969_at	4833403J16Rik	1.0	**3.8**
1419866_s_at	Atxn2	1.0	**3.8**
1420535_a_at	Nub1	−1.0	**3.7**
1437801_at	Morf4l1	−1.0	**3.7**
1438009_at	Hist1h2ad	1.6	**3.6**
1435749_at	Gda	1.9	**3.6**
1439005_x_at	Ywhaz	−1.1	**3.6**

Table 1. BALB/c mice were intranasally infected with 5 × 10^5^ CFU *B. bronchiseptica* or treated with PBS. Mice were sacrificed 7 or 42 days post infection. Splenocytes from both infected and control mice were isolated and pooled (*n =* 6 per group), and CD4^+^CD25^+^ T cells were flow cytometrically sorted, followed by RNA preparation. Samples were analyzed on whole transcriptome microarrays. Fold changes of differential gene regulation were calculated for each time point, comparing CD4^+^CD25^+^ T cells from infected mice vs. CD4^+^CD25^+^ T cells from the respective uninfected controls. Same was performed for CD4^+^CD25^−^ T cells. Genes for which a fold change regulation of more than 2-fold (up or down) was detected, either 7 days or 42 days post infection, in CD4^+^CD25^+^ but not CD4^+^CD25^−^ T cells, were ranked according to their fold change transcriptional regulation on Day 42. The table represents the top 30 up- and down-regulated genes of this ranked list.

**Table 2:** Comparison of gene expression between CD4^+^CD25^+^ and CD4^+^CD25^−^ T cells reveals T_reg_ signature.

Probe Set ID	Gene Symbol	CD25^+^ vs. CD25^−^			
		contr.		Bb inf.	
		Day 7	Day 42	Day 7	Day 42
1420692_at	Il2ra	53.8	14.7	29.7	**25.1**
1420765_a_at	Foxp3	39.9	11.9	32.0	**16.2**
1439569_at	Gpr83	15.3	11.3	10.7	**14.6**
1429159_at	Itih5	16.5	7.0	8.3	**13.9**
1418507_s_at	Socs2	12.5	4.8	13.0	**13.6**
1447541_s_at	Itgae	27.8	10.7	20.7	**13.1**
1434141_at	Gucy1a3	5.4	4.5	8.6	**13.1**
1457342_at	Ikzf4	10.5	9.9	8.5	**13.0**
1456956_at	Ikzf2	22.6	13.7	11.7	**12.8**
1419334_at	Ctla4	12.8	9.9	9.5	**12.1**
1448943_at	Nrp1	12.6	5.9	6.3	**11.4**
1429918_at	Arhgap20	9.2	8.8	9.0	**10.9**
1449216_at	Itgae	16.1	8.4	15.0	**10.5**
1437542_at	Ikzf2	15.2	8.4	10.7	**10.4**
1423626_at	Dst	9.0	7.4	5.9	**9.4**
1418084_at	Nrp1	11.8	5.6	8.2	**8.8**
1425145_at	Il1rl1	12.0	4.1	11.0	**8.5**
1448944_at	Nrp1	7.6	4.8	5.3	**7.8**
1426208_x_at	Plagl1	13.6	8.1	9.5	**7.7**
1438274_at	Ikzf4	8.0	7.5	6.3	**7.7**
1428329_a_at	Ift80	3.9	4.4	3.5	**7.6**
1428074_at	Tmem158	5.0	3.2	4.3	**7.1**
1449109_at	Socs2	7.9	4.2	8.4	**6.8**
1423415_at	Gpr83	8.3	11.6	8.0	**6.8**
1417601_at	Rgs1	2.7	4.9	3.0	**6.4**
1420788_at	Klrg1	13.2	5.3	10.0	**6.4**
1419219_at	Cyp4f18	12.2	10.3	8.6	**6.2**
1457198_at	Nrp1	7.7	6.7	6.1	**5.9**
1460469_at	Tnfrsf9	10.9	5.0	6.9	**5.8**
1418057_at	Tiam1	8.3	4.1	5.5	**5.5**

Table 2. BALB/c mice were intranasally infected with 5 × 10^5^ CFU *B. bronchiseptica* (Bb) or treated with PBS. Mice were sacrificed 7 or 42 days post infection. Splenocytes from both infected and control mice were isolated and pooled (*n =* 6 per group), and CD4^+^CD25^+^ as well as CD4^+^CD25^−^ T cells were flow cytometrically sorted, followed by RNA preparation. Samples were analyzed on whole transcriptome microarrays. Fold changes of differential gene regulation were calculated for each time point, comparing CD4^+^CD25^+^ T cells vs. CD4^+^CD25^−^ T cells from all four conditions. Genes with a fold change of more than 2-fold in all four conditions were ranked in descending order, according to their fold change in the CD4^+^CD25^+^ vs. CD4^+^CD25^−^ comparison on Day 42 post infection. The table shows the top 30 up-regulated Probe Set IDs of this list.

## Supplementary Materials

The following are available online at http://www.mdpi.com/1422-0067/19/9/2602/s1.

## Abbreviations

MDPI	Multidisciplinary Digital Publishing Institute
DOAJ	Directory of open access journals

## References

[1] Dickson R.P., Erb-Downward J.R., Martinez F.J., Huffnagle G.B. (2016). The microbiome and the respiratory tract. Annu. Rev. Physiol..

[2] Wu B.G., Segal L.N. (2017). Lung microbiota and its impact on the mucosal immune phenotype. Microbiol. Spectr..

[3] Chen K., Kolls J.K. (2013). T cell-mediated host immune defenses in the lung. Annu. Rev. Immunol..

[4] Duan W., Croft M. (2014). Control of regulatory T cells and airway tolerance by lung macrophages and dendritic cells. Ann. Am. Thorac. Soc..

[5] Hasenberg M., Stegemann-Koniszewski S., Gunzer M. (2013). Cellular immune reactions in the lung. Immunol. Rev..

[6] Weitnauer M., Mijosek V., Dalpke A.H. (2016). Control of local immunity by airway epithelial cells. Mucosal Immunol..

[7] Garbi N., Lambrecht B.N. (2017). Location, function, and ontogeny of pulmonary macrophages during the steady state. Pflugers Arch..

[8] Kolahian S., Oz H.H., Zhou B., Griessinger C.M., Rieber N., Hartl D. (2016). The emerging role of myeloid-derived suppressor cells in lung diseases. Eur. Respir. J..

[9] Garib F.Y., Rizopulu A.P. (2015). T-regulatory cells as part of strategy of immune evasion by pathogens. Biochemistry (Mosc.).

[10] Cyktor J.C., Turner J. (2011). Interleukin-10 and immunity against prokaryotic and eukaryotic intracellular pathogens. Infect. Immun..

[11] Levast B., Li Z., Madrenas J. (2015). The role of IL-10 in microbiome-associated immune modulation and disease tolerance. Cytokine.

[12] Bisgaard H., Hermansen M.N., Buchvald F., Loland L., Halkjaer L.B., Bonnelykke K., Brasholt M., Heltberg A., Vissing N.H., Thorsen S.V. (2007). Childhood asthma after bacterial colonization of the airway in neonates. N. Engl. J. Med..

[13] Taylor S.L., Wesselingh S., Rogers G.B. (2016). Host-microbiome interactions in acute and chronic respiratory infections. Cell. Microbiol..

[14] Von Linstow M.L., Schonning K., Hoegh A.M., Sevelsted A., Vissing N.H., Bisgaard H. (2013). Neonatal airway colonization is associated with troublesome lung symptoms in infants. Am. J. Respir. Crit. Care Med..

[15] McAleer J.P., Kolls J.K. (2018). Contributions of the intestinal microbiome in lung immunity. Eur. J. Immunol..

[16] Samuelson D.R., Welsh D.A., Shellito J.E. (2015). Regulation of lung immunity and host defense by the intestinal microbiota. Front. Microbiol..

[17] Liu Y.Y., Chiang C.H., Chuang C.H., Liu S.L., Jheng Y.H., Ryu J.H. (2014). Spillover of cytokines and reactive oxygen species in ventilator-induced lung injury associated with inflammation and apoptosis in distal organs. Respir. Care.

[18] Rahman Fink N., Chawes B.L., Thorsen J., Stokholm J., Krogfelt K., Schjorring S., Kragh M., Bonnelykke K., Brix S. (2018). Neonates colonized with pathogenic bacteria in the airways have a low-grade systemic inflammation. Allergy.

[19] Teichert T., Vossoughi M., Vierkotter A., Sugiri D., Schikowski T., Hoffmann B., Schulte T., Roden M., Raulf-Heimsoth M., Luckhaus C. (2014). Investigating the spill-over hypothesis: Analysis of the association between local inflammatory markers in sputum and systemic inflammatory mediators in plasma. Environ. Res..

[20] Yevsa T., Ebensen T., Fuchs B., Zygmunt B., Libanova R., Gross R., Schulze K., Guzman C.A. (2013). Development and characterization of attenuated metabolic mutants of *Bordetella bronchiseptica* for applications in vaccinology. Environ. Microbiol..

[21] Melvin J.A., Scheller E.V., Miller J.F., Cotter P.A. (2014). *Bordetella pertussis* pathogenesis: Current and future challenges. Nat. Rev. Microbiol..

[22] Cotter P.A., Jones A.M. (2003). Phosphorelay control of virulence gene expression in *Bordetella*. Trends Microbiol..

[23] Trainor E.A., Nicholson T.L., Merkel T.J. (2015). *Bordetella pertussis* transmission. Pathog. Dis..

[24] Hewlett E.L., Burns D.L., Cotter P.A., Harvill E.T., Merkel T.J., Quinn C.P., Stibitz E.S. (2014). Pertussis pathogenesis—What we know and what we don’t know. J. Infect. Dis..

[25] Banemann A., Gross R. (1997). Phase variation affects long-term survival of *Bordetella bronchiseptica* in professional phagocytes. Infect. Immun..

[26] Forde C.B., Parton R., Coote J.G. (1998). Bioluminescence as a reporter of intracellular survival of *Bordetella bronchiseptica* in murine phagocytes. Infect. Immun..

[27] Buboltz A.M., Nicholson T.L., Weyrich L.S., Harvill E.T. (2009). Role of the type III secretion system in a hypervirulent lineage of *Bordetella bronchiseptica*. Infect. Immun..

[28] Mann P., Goebel E., Barbarich J., Pilione M., Kennett M., Harvill E. (2007). Use of a genetically defined double mutant strain of *Bordetella bronchiseptica* lacking adenylate cyclase and type III secretion as a live vaccine. Infect. Immun..

[29] Weyrich L.S., Rolin O.Y., Muse S.J., Park J., Spidale N., Kennett M.J., Hester S.E., Chen C., Dudley E.G., Harvill E.T. (2012). A type VI secretion system encoding locus is required for *Bordetella bronchiseptica* immunomodulation and persistence in vivo. PLoS ONE.

[30] Reissinger A., Skinner J.A., Yuk M.H. (2005). Downregulation of mitogen-activated protein kinases by the *Bordetella bronchiseptica* Type III secretion system leads to attenuated nonclassical macrophage activation. Infect. Immun..

[31] Siciliano N.A., Skinner J.A., Yuk M.H. (2006). *Bordetella bronchiseptica* modulates macrophage phenotype leading to the inhibition of CD4+ T cell proliferation and the initiation of a Th17 immune response. J. Immunol..

[32] Guzman C.A., Rohde M., Bock M., Timmis K.N. (1994). Invasion and intracellular survival of *Bordetella bronchiseptica* in mouse dendritic cells. Infect. Immun..

[33] Gueirard P., Ave P., Balazuc A.M., Thiberge S., Huerre M., Milon G., Guiso N. (2003). *Bordetella bronchiseptica* persists in the nasal cavities of mice and triggers early delivery of dendritic cells in the lymph nodes draining the lower and upper respiratory Tract. Infect. Immun..

[34] Nagamatsu K., Kuwae A., Konaka T., Nagai S., Yoshida S., Eguchi M., Watanabe M., Mimuro H., Koyasu S., Abe A. (2009). *Bordetella evades* the host immune system by inducing IL-10 through a type III effector, BopN. J. Exp. Med..

[35] Pilione M.R., Harvill E.T. (2006). The *Bordetella bronchiseptica* type III secretion system inhibits gamma interferon production that is required for efficient antibody-mediated bacterial clearance. Infect. Immun..

[36] Skinner J.A., Pilione M.R., Shen H., Harvill E.T., Yuk M.H. (2005). *Bordetella* type III secretion modulates dendritic cell migration resulting in immunosuppression and bacterial persistence. J. Immunol..

[37] Bruder D., Probst-Kepper M., Westendorf A.M., Geffers R., Beissert S., Loser K., von Boehmer H., Buer J., Hansen W. (2004). Neuropilin-1: A surface marker of regulatory T cells. Eur. J. Immunol..

[38] Hansen W., Loser K., Westendorf A.M., Bruder D., Pfoertner S., Siewert C., Huehn J., Beissert S., Buer J. (2006). G protein-coupled receptor 83 overexpression in naive CD4^+^CD25^−^ T cells leads to the induction of Foxp3^+^ regulatory T cells in vivo. J. Immunol..

[39] Sugimoto N., Oida T., Hirota K., Nakamura K., Nomura T., Uchiyama T., Sakaguchi S. (2006). Foxp3-dependent and -independent molecules specific for CD25+CD4+ natural regulatory T cells revealed by DNA microarray analysis. Int. Immunol..

[40] Wing K., Onishi Y., Prieto-Martin P., Yamaguchi T., Miyara M., Fehervari Z., Nomura T., Sakaguchi S. (2008). CTLA-4 control over Foxp3+ regulatory T cell function. Science.

[41] Lehmann J., Huehn J., de la Rosa M., Maszyna F., Kretschmer U., Krenn V., Brunner M., Scheffold A., Hamann A. (2002). Expression of the integrin alpha Ebeta 7 identifies unique subsets of CD25^+^ as well as CD25^−^ regulatory T cells. Proc. Natl. Acad. Sci. USA.

[42] Pan F., Yu H., Dang E.V., Barbi J., Pan X., Grosso J.F., Jinasena D., Sharma S.M., McCadden E.M., Getnet D. (2009). Eos mediates Foxp3-dependent gene silencing in CD4+ regulatory T cells. Science.

[43] Hori S., Nomura T., Sakaguchi S. (2003). Control of regulatory T cell development by the transcription factor Foxp3. Science.

[44] Kim Y.G. (2017). Microbiota influences vaccine and mucosal adjuvant efficacy. Immune Netw..

[45] Lynn D.J., Pulendran B. (2018). The potential of the microbiota to influence vaccine responses. J. Leukoc. Biol..

[46] Nguyen Q.N., Himes J.E., Martinez D.R., Permar S.R. (2016). The impact of the gut microbiota on humoral immunity to pathogens and vaccination in early infancy. PLoS Pathog..

[47] Zimmermann P., Curtis N. (2018). The influence of probiotics on vaccine responses—A systematic review. Vaccine.

[48] Braciale T.J., Sun J., Kim T.S. (2012). Regulating the adaptive immune response to respiratory virus infection. Nat. Rev. Immunol..

[49] McGill J., Heusel J.W., Legge K.L. (2009). Innate immune control and regulation of influenza virus infections. J. Leukoc. Biol..

[50] Wilder M.S., Sword C.P. (1967). Mechanisms of pathogenesis in *Listeria monocytogenes* infection. II. Characterization of listeriosis in the CD-1 mouse and survey of biochemical lesions. J. Bacteriol..

[51] Calame D.G., Mueller-Ortiz S.L., Wetsel R.A. (2016). Innate and adaptive immunologic functions of complement in the host response to *Listeria monocytogenes* infection. Immunobiology.

[52] McGuirk P., McCann C., Mills K.H.G. (2002). Pathogen-specific T regulatory 1 cells induced in the respiratory tract by a bacterial molecule that stimulates interleukin 10 production by dendritic Cells. J. Exp. Med..

[53] Moreno G., Errea A., Van Maele L., Roberts R., Léger H., Sirard J.C., Benecke A., Rumbo M., Hozbor D. (2013). Toll-like receptor 4 orchestrates neutrophil recruitment into airways during the first hours of *Bordetella pertussis* infection. Microbes Infect..

[54] Rolin O., Smallridge W., Henry M., Goodfield L., Place D., Harvill E.T. (2014). Toll-like receptor 4 limits transmission of *Bordetella bronchiseptica*. PLoS ONE.

[55] Zanin-Zhorov A., Tal-Lapidot G., Cahalon L., Cohen-Sfady M., Pevsner-Fischer M., Lider O., Cohen I.R. (2007). Cutting edge: T cells respond to lipopolysaccharide innately via TLR4 signaling. J. Immunol..

[56] McGuirk P., Mills K.H. (2000). A regulatory role for interleukin 4 in differential inflammatory responses in the lung following infection of mice primed with Th1- or Th2-inducing pertussis vaccines. Infect. Immun..

[57] Glinka Y., Prud’homme G.J. (2008). Neuropilin-1 is a receptor for transforming growth factor beta-1, activates its latent form, and promotes regulatory T cell activity. J. Leukoc. Biol..

[58] Gerondakis S., Siebenlist U. (2010). Roles of the NF-kappaB pathway in lymphocyte development and function. Cold Spring Harb. Perspect. Biol..

[59] Tiemessen M.M., Jagger A.L., Evans H.G., van Herwijnen M.J., John S., Taams L.S. (2007). CD4^+^CD25^+^Foxp3^+^ regulatory T cells induce alternative activation of human monocytes/macrophages. Proc. Natl. Acad. Sci. USA.

[60] Przemska-Kosicka A., Childs C.E., Maidens C., Dong H., Todd S., Gosney M.A., Tuohy K.M., Yaqoob P. (2018). Age-related changes in the natural killer cell response to seasonal influenza vaccination are not influenced by a synbiotic: A randomised controlled trial. Front. Immunol..

[61] Betts R.J., Prabhu N., Ho A.W., Lew F.C., Hutchinson P.E., Rotzschke O., Macary P.A., Kemeny D.M. (2012). Influenza A virus infection results in a robust, antigen-responsive, and widely disseminated Foxp3+ regulatory T cell response. J. Virol..

[62] Vissing N.H., Chawes B.L., Bisgaard H. (2013). Increased risk of pneumonia and bronchiolitis after bacterial colonization of the airways as neonates. Am. J. Respir. Crit. Care Med..

[63] Brockmeier S.L. (2004). Prior infection with *Bordetella bronchiseptica* increases nasal colonization by *Haemophilus parasuis* in swine. Vet. Microbiol..

[64] Loving C.L., Brockmeier S.L., Vincent A.L., Palmer M.V., Sacco R.E., Nicholson T.L. (2010). Influenza virus coinfection with *Bordetella bronchiseptica* enhances bacterial colonization and host responses exacerbating pulmonary lesions. Microb. Pathog..

[65] Basha S., Surendran N., Pichichero M. (2014). Immune responses in neonates. Expert Rev. Clin. Immunol..

[66] Lynch S.V. (2016). The lung microbiome and airway disease. Ann. Am. Thorac. Soc..

[67] Viegas N., Andzinski L., Wu C.F., Komoll R.M., Gekara N., Dittmar K.E., Weiss S., Jablonska J. (2013). IFN-gamma production by CD27(+) NK cells exacerbates *Listeria monocytogenes* infection in mice by inhibiting granulocyte mobilization. Eur. J. Immunol..

[68] Kirberg J., Baron A., Jakob S., Rolink A., Karjalainen K., von Boehmer H. (1994). Thymic selection of CD8+ single positive cells with a class II major histocompatibility complex-restricted receptor. J. Exp. Med..

[69] Morgan D.J., Liblau R., Scott B., Fleck S., McDevitt H.O., Sarvetnick N., Lo D., Sherman L.A. (1996). CD8(+) T cell-mediated spontaneous diabetes in neonatal mice. J. Immunol..

[70] Arico B., Rappuoli R. (1987). *Bordetella parapertussis* and *Bordetella bronchiseptica* contain transcriptionally silent pertussis toxin genes. J. Bacteriol..

[71] Stegemann S., Dahlberg S., Kroger A., Gereke M., Bruder D., Henriques-Normark B., Gunzer M. (2009). Increased susceptibility for superinfection with Streptococcus pneumoniae during influenza virus infection is not caused by TLR7-mediated lymphopenia. PLoS ONE.

[72] Bindea G., Mlecnik B., Hackl H., Charoentong P., Tosolini M., Kirilovsky A., Fridman W.H., Pages F., Trajanoski Z., Galon J. (2009). ClueGO: A Cytoscape plug-in to decipher functionally grouped gene ontology and pathway annotation networks. Bioinformatics.

