# Supplementary material for: Respiratory Bordetella bronchiseptica Carriage is Associated with Broad Phenotypic Alterations of Peripheral CD4+CD25+ T Cells and Differentially Affects Immune Responses to Secondary Non-Infectious and Infectious Stimuli in Mice
Source: Int J Mol Sci. 2018 Sep 1;19(9):2602. doi: 10.3390/ijms19092602 (PMC6165163; doi:10.3390/ijms19092602)
Supplement: Supplementary file 1 [file ijms-19-02602-s001.pdf]

International Journal of
Molecular Sciences

MDPI

Article

Respiratory *Bordetella bronchiseptica* Carriage is Associated with Broad Phenotypic Alterations of Peripheral CD4+CD25+ T Cells and Differentially Affects Immune Responses to Secondary Non-Infectious and Infectious Stimuli in Mice

Andreas Jeron, Julia D. Boehme, Julia Volckmar, Marcus Gereke, Tetyana Yevsa, Robert Geffers, Carlos A. Guzmán, Jens Schreiber, Sabine Stegemann-Koniszewski and Dunja Bruder

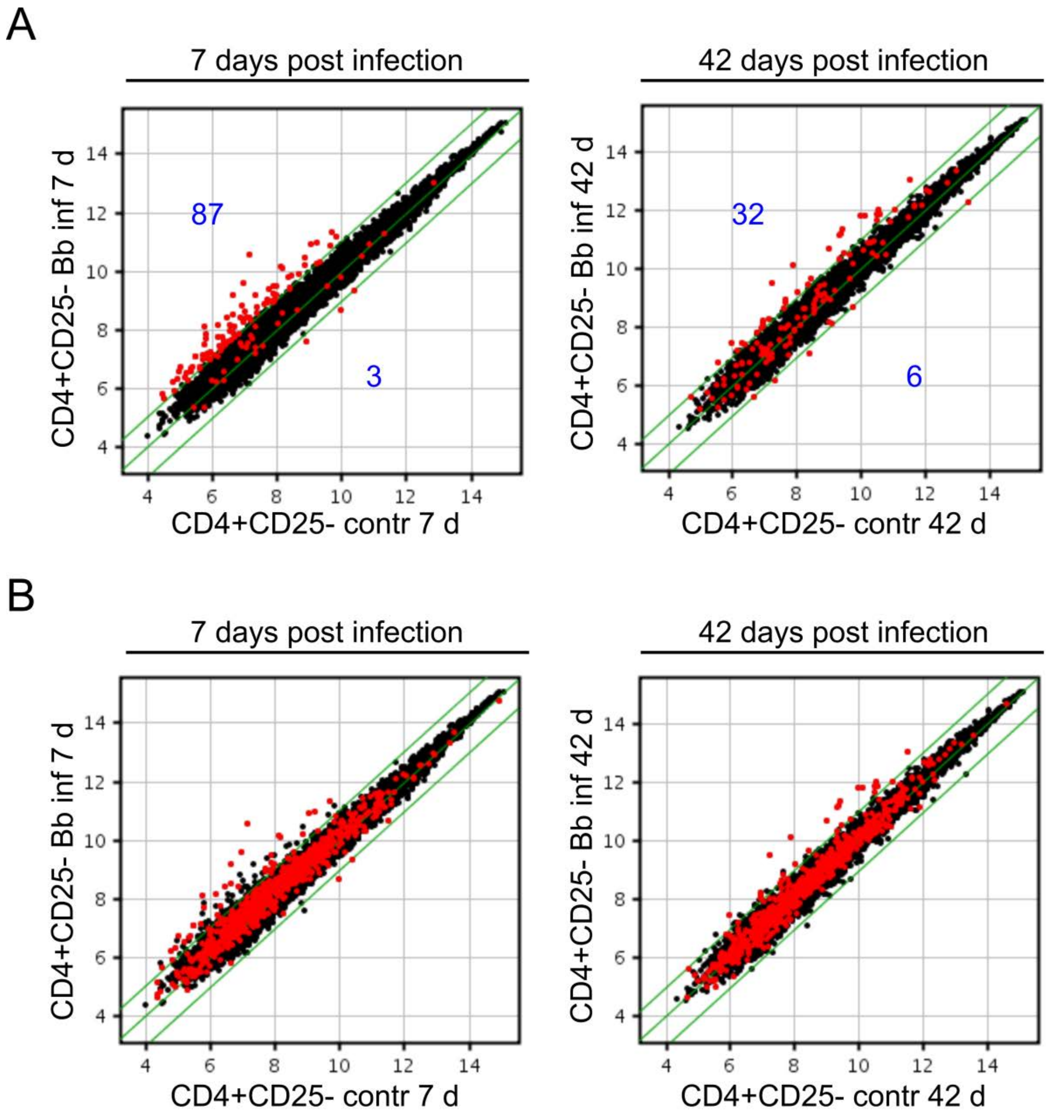

Figure S1. Differential gene expression of conventional $CD4^+CD25^-$ T cells isolated from *B. bronchiseptica* infected mice, on day 7 or day 42 post infection. BALB/c mice were intranasally infected with $5x10^5$ CFU *B. bronchiseptica* (Bb) or treated with PBS (contr). Mice were sacrificed 7 or 42 days post infection. Splenocytes from both infected and control mice were isolated and pooled (n=6 per group), and $CD4^+CD25^-$ T cells were flow cytometrically sorted for RNA preparation.

Samples were analyzed on whole transcriptome microarrays. Fold changes of differential gene regulation were calculated for each time point, comparing CD4$^+$CD25$^-$ T cells from infected mice vs. CD4$^+$CD25$^-$ T cells from uninfected controls. For indicated data comparisons, scatter dot plots representing log$_2$ normalized signal intensities are shown. Green lines indicate the bisecting line and fold change criterions (more/less than two-fold). (a) Genes fulfilling the fold change criterion on day 7 or day 42 post infection are marked in red. Numbers of regulated genes are indicated in blue. (b) Genes found to be regulated more/less than two-fold in the microarray analysis of CD4$^+$CD25$^+$ T cells from *B. bronchiseptica* infected mice isolated on day 7 or day 42 post infection, correlating to Figure 3D, are marked in red.

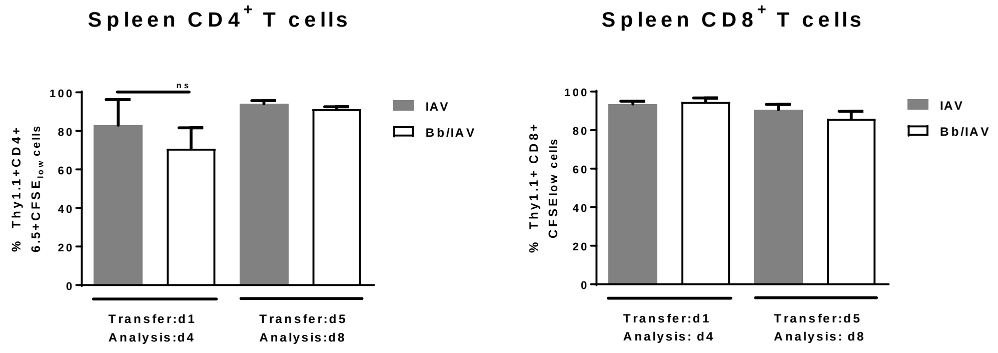

Figure S2. Dissemination of influenza-specific T cells to the spleen. BALB/c mice were intranasally infected with 1x10^6 CFU *B. bronchiseptica* (Bb) or left uninfected followed by intranasal infection of all mice with 0.04 LD$_{50}$ IAV PR8 on day 42. Naive CD4$^+$Thy1.1$^+$ or CD8$^+$Thy1.1$^+$ T lymphocytes isolated from spleens and cervical lymph nodes of TCR-HA mice (CD4$^+$ T cells) or CL4 mice (CD8$^+$ T cells), were labelled with carboxyfluorescein diacetate succinimidyl ester (CFSE). Cells were adoptively transferred into infected mice, on day 1 or day 5 post IAV infection. On day 3 following adoptive transfer, lymphocytes were recovered from the spleen and proliferation was determined by the analysis of CFSE dilution. Bar graphs show the % proliferated (CFSE$_{low}$) Thy1.1$^+$CD4$^+$ or Thy1.1$^+$CD8$^+$ T cells. Bars represent the mean ±SEM/group and data are shown for one out of two independent experiments (n = 4–6 mice/group). Groups were compared using the two-tailed Mann-Whitney test.

Table S1. Gene symbols and fold-change regulation of Gene Ontology (GO)-term content displayed in figure 4. The 13 enriched GO-term groups displayed in figure 4 are listed, and for each group, up- and down-regulated transcripts are included together with their fold-change (FC) of regulation. FC regulation, refers to transcriptional regulation in CD4$^+$CD25$^+$ T cells isolated from *B. bronchiseptica* carriers, compared to CD4$^+$CD25$^+$ T cells isolated from controls. The lists for the different GO-term groups, are limited to the top-10 up- and/or down-regulated genes, where their number exceeds 10.

**transcription, DNA-templated**	**FC**	**negative regulation of RNA metabolic process**	**FC**	**leukocyte differentiation**	**FC**	**negative regulation of mRNA metabolic process**	**FC**	**regulation of intracellular protein transport**	**FC**
Cdk12	2.1	Irf2	4.7	Ltf	4.1	S100a9	7.3	Pkig	3.6
Kat6a	2.1	Elavl1	4.5	Fnip1	4.0	Fxr1	5.3	Cdc42	2.8
Kmt2e	2.4	Strap	4.3	Foxp1	2.8	Elavl1	4.5	Tmem30a	2.8
Srsf1	2.7	Fnip1	4.0	Hsp90aa1	2.8	Dyrk1a	3.6	Pik3r1	2.6
Irf2	4.7	Fli1	3.7	Lig4	2.8	Dkc1	3.5	Tlr4	2.6
Strap	4.3	Morf4l1	3.7	Rora	2.8	Exosc8	3.3	Erlec1	2.4
Ltf	4.1	Dyrk1a	3.6	Braf	2.7	Rps27l	3.3	F2r	2.4
Fnip1	4.0	Pkig	3.6	Egr3	2.7	Ncl	2.9	Ube2d3	2.4
Tax1bp1	3.9	Dkc1	3.5	Bcl11a	2.6	Rnf20	2.9	Ugcg	2.4
Fli1	3.7	Hsph1	3.5	Ep300	2.6	Pum2	2.7	Mief1	2.3

Rnf14	-2.8	Boll	-2.6	Hcls1	-2.3	Exosc5	-2.9	Hcls1	-2.3
Ptbp1	-2.5	Ptbp1	-2.5	Satb1	-2.3	Boll	-2.6		
Nrip1	-2.5	Nrip1	-2.5	Tnfaip3	-2.2	Ptbp1	-2.5		
Satb1	-2.3	Satb1	-2.3			Dcps	-2.3		
Hcls1	-2.3	Hcls1	-2.3			Snrnp70	-2.2		
Tnfaip3	-2.2	Ptprk	-2.2			Zfp36	-2.1		
Snrnp70	-2.2	Zfp36	-2.1						
Ptprk	-2.2								
Zfp775	-2.1								
Zfp36	-2.1								

**RNA splicing**	**FC**	**myeloid cell differentiation**	**FC**	**protein K48-linked ubiquitination**	**FC**	**protein deacetylation**	**FC**	**regulation of actomyosin structure organization**	**FC**
Strap	4.3	Hbb-b2	4.3	G2e3	3.0	Morf4l1	3.7	Cdc42	2.8
Dyrk1a	3.6	Ltf	4.1	Rnf20	2.9	Dyrk1a	3.6	Sdc4	2.8
Esrp2	3.5	Fli1	3.7	Ube2d1	2.6	Atxn3	3.0	Braf	2.7
Rbm39	3.5	Hba-a1	3.1	Cdc27	2.4	Ep300	2.6	Pik3r1	2.6
Psip1	3.0	Foxp1	2.8	March1	2.4	Hdac2	2.3	Pfn1	2.4
Rbmx2	2.7	Ep300	2.6	Ube2d3	2.4	Tbl1xr1	2.3	Hdac2	2.3
Srsf1	2.7	Pik3r1	2.6	Peli1	2.3	Lrrk2	2.2	Mef2c	2.3
Pik3r1	2.6	Sp3	2.6	Rnf6	2.3	Rest	2.1	S100a10	2.3
Prpf40a	2.5	Kmt2e	2.4	Ube2d2a	2.2	Sap30	2.1	Rdx	2.2
Slu7	2.4	Prtn3	2.4	Ube3a	2.1	Sfpq	2.1	Rock1	2.2
Snrnp70	-2.2	Zfp36	-2.1	Tnfaip3	-2.2				
Dcps	-2.3	Hcls1	-2.3	Rnf14	-2.8				
Ptbp1	-2.5								

**intracellular steroid hormone receptor signaling pathway**	**FC**	**ncRNA 3'-end processing**	**FC**	**cytoplasmic pattern recognition receptor signaling pathway**	**FC**
Foxp1	2.8	Dkc1	3.5	Pum2	2.7
Ep300	2.6	Exosc8	3.3	Tlr4	2.6
Ar	2.3	Rnf20	2.9	Ankrd17	2.2
Rnf6	2.3	Ssb	2.3	Riok3	2.2
Strn3	2.2	Eri1	2.1	Nfkbia	2.1
Cry1	2.1	Fip1l1	2.1	Tnfaip3	-2.2
Ptges3	2.1	Fbl	-2.4		
Uba5	2.1	Exosc5	-2.9		
Ube3a	2.1				
Rnf14	-2.8				
